# Mechanically interlocked monolayer and bilayer two-dimensional polymers with high elastic modulus

**DOI:** 10.1038/s44160-025-00930-4

**Published:** 2025-11-13

**Authors:** Ye Yang, André Knapp, David Bodesheim, Alexander Croy, Mike Hambsch, Ilka Hermes, Chandrasekhar Naisa, Darius Pohl, Bernd Rellinghaus, Changsheng Zhao, Stefan C. B. Mannsfeld, Gianaurelio Cuniberti, Zhiyong Wang, Renhao Dong, Andreas Fery, Xinliang Feng

**Affiliations:** 1 Center for Advancing Electronics Dresden and Faculty of Chemistry and Food Chemistry, TUD Dresden University of Technology, Dresden, Germany; 2 Institute of Physical Chemistry and Polymer Physics, Leibniz Institute of Polymer Research Dresden, Dresden, Germany; 3 Institute for Materials Science and Max Bergmann Center for Biomaterials, TUD Dresden University of Technology, Dresden, Germany; 4 Institute of Physical Chemistry, Friedrich Schiller University Jena, Jena, Germany; 5 Center for Advancing Electronics Dresden and Faculty of Electrical and Computer Engineering, TUD Dresden University of Technology, Dresden, Germany; 6 Max Planck Institute of Microstructure Physics, Halle (Saale), Germany; 7 Dresden Center for Nanoanalysis (DCN), Center for Advancing Electronics Dresden, TUD Dresden University of Technology, Dresden, Germany; 8 College of Polymer Science and Engineering, State Key Laboratory of Polymer Materials Engineering, Sichuan University, Chengdu, China; 9 Dresden Center for Computational Materials Science (DCMS), TUD Dresden University of Technology, Dresden, Germany; 10 Department of Chemistry, The University of Hong Kong, Hong Kong, China; 11 Materials Innovation Institute for Life Sciences and Energy (MILES), HKU-SIRI, Shenzhen, China; 12 Physical Chemistry of Polymeric Materials, TUD Dresden University of Technology, Dresden, Germany

**Keywords:** Polymer synthesis, Mechanical properties

## Abstract

Two-dimensional polymers (2DPs), comprising mono- or multilayer covalent polymeric networks with long-range order in two orthogonal directions, are of considerable interest due to their unique physicochemical properties. However, achieving precise thickness control from monolayer to bilayer, crucial for exploring proximity effect-driven phenomena beyond the monolayer limit, remains synthetically challenging. Here we report the on-water surface synthesis of crystalline mechanically interlocked monolayer and bilayer 2DP (MI-M2DP and MI-B2DP) films by embedding macrocyclic molecules with one and two cavities into 2DP backbones. The incorporation of bulky macrocyclic molecules introduces periodic mechanical bonds that precisely control interlayer interlocking, enabling selective monolayer or bilayer 2DP formation. Both MI-M2DP and MI-B2DP exhibit homogeneous, large-area films with ordered hexagonal pores and high modulus. MI-B2DP demonstrates an exceptionally high effective Young’s modulus of 151 ± 16 GPa (indentation method), surpassing MI-M2DP (90 ± 14 GPa), van der Waals-stacked MI-M2DPs (46 ± 11 GPa) and other reported multilayer 2DPs (<50 GPa). Modelling confirms that the mechanical interlocking minimizes interlayer sliding and reinforces the structure.

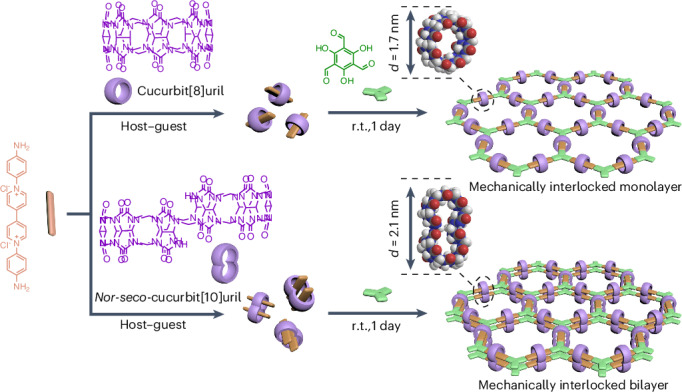

## Abstract (web summary)

Mechanically interlocked monolayer and bilayer two-dimensional polymers (2DPs) are synthesized on the water surface by embedding macrocyclic molecules with one and two cavities into the backbones. The resulting bilayer 2DP displays a high effective Young’s modulus, exceeding other reported multilayer 2DPs.

## Main

Two-dimensional polymers (2DPs) and their layer-stacked covalent organic frameworks (2D COFs) are a class of crystalline, layered materials with periodic extension in two orthogonal directions^1^. In these organic 2D crystal materials, adjacent layers are predominantly held together by weak van der Waals (vdW) forces, *π*–*π* interactions or hydrogen bonding^2,3^. While extensive studies have focused on their in-plane structure and functionality^4^, the manner in which these atomically thin layers interact across the third dimension introduces a powerful, yet underexplored tunability^5^. Specifically, controlled stacking into a bilayer can unlock emergent physical and chemical phenomena distinct from those of the monolayer limit, arising from the proximity effect, such as interlayer electronic coupling, and symmetry breaking at the interface^6^. For instance, introducing a small rotational misalignment between stacked layers enables moiré superlattices with spatially modulated electronic or excitonic landscapes^7,8^, while layer stacking can reduce the band gap or generate new states near the Fermi level^9,10^. Likewise, the mechanical response of bilayer 2DPs often diverges from that of their monolayer counterparts: weak interlayer interactions can facilitate decoupling or sliding, diminishing overall mechanical strength^11–13^. These phenomena highlight the importance of constructing monolayer and bilayer 2DPs to explore interlayer structure–property relationships and enable rational materials design^14^. Top-down approaches, including physical^15^ and chemical^16^ exfoliation, can yield ultrathin layers, but often suffer from poor structural integrity and non-uniform thickness^17^. In contrast, bottom-up strategies, such as on-surface synthesis^18^ and Langmuir–Blodgett (LB) techniques^19^, have demonstrated success in producing monolayer 2DPs. However, extending these strategies to bilayers, whether via direct synthesis^20^ or layer-by-layer transfer^21^, invariably disrupts structural uniformity because interlayer interactions and offsets introduce disorder. Therefore, precise control over 2DP thickness from monolayer to bilayer—while preserving well-defined in-plane structures—remains a synthetic challenge.

In this study, we demonstrated the on-water surface synthesis of crystalline mechanically interlocked monolayer (**MI-M2DP**) and bilayer (**MI-B2DP**) 2DP films by incorporating macrocyclic molecules (MCMs) into the backbones. Through cooperative host–guest assembly between MCMs (cucurbit[8]uril (**CB8**) with a single cavity or *nor-seco*-cucurbit[10]uril (***ns*-CB10**) with double cavities) and 1,1′-bis(4-aminophenyl)-[4,4′-bipyridine]-1,1′-diium chloride (**V-2NH**_**2**_) molecules, we achieved precise control over the number of interlocking layers. The resulting films were characterized by imaging and X-ray scattering techniques, confirming their crystallinity. Using strain-induced elastic buckling instability for mechanical measurements (SIEBIMM) and atomic force microscopy (AFM) nanoindentation techniques^22,23^, the effective Young’s modulus (*E*_Young_) of **MI-M2DP** and **MI-B2DP** were systematically examined, showing ultrahigh elastic modulus. Theoretical calculations were conducted to elucidate the underlying mechanism governing the observed layer-dependent mechanical behaviour. The **MI-B2DP** film was further integrated as the membrane for seawater desalination to demonstrate its practical utility. This study sheds light on the controlled synthesis of crystalline 2DPs at the monolayer or bilayer level and provides potential avenues to address the challenges of exploring the interlayer structure–property relationships.

## Results

### Design principle and on-water surface synthesis

MCMs^24^ are of interest as supramolecular scaffolds for constructing linear polymers and crosslinked polymer networks through host–guest chemistry^25^. A key feature of MCMs is their pronounced steric bulk, which disrupts *π*–*π* stacking between adjacent polymer backbones. This characteristic presents a unique opportunity to suppress layer stacking in 2DPs and enables structural control in the out-of-plane direction. Leveraging this property, we propose that MCMs containing one or more host cavities could serve as programmable spacers to regulate interlayer interactions and guide the synthesis of interlocked 2DPs with defined layer numbers and in-plane periodicity^26^. To explore this concept, we employed cucurbiturils as model MCMs. **CB8**, which features a single host cavity, was used to suppress interlayer interaction and confine 2D polymerization to a monolayer (**MI-M2DP**)^27^. For bilayer formation, we designed and synthesized ***ns*-CB10**^28,29^ via the condensation between glycoluril and formaldehyde (Supplementary Figs. 1–3). The resulting ***ns*-CB10** with two adjacent cavities (∼6.5 Å diameter; Supplementary Fig. 4) is capable of hosting two guest molecules. This dual-cavity architecture allows for precise spatial alignment of monomeric units across two stacked layers, thereby offering a molecular-level design principle for constructing bilayer 2DP (**MI-B2DP**).

**Fig. 1:**
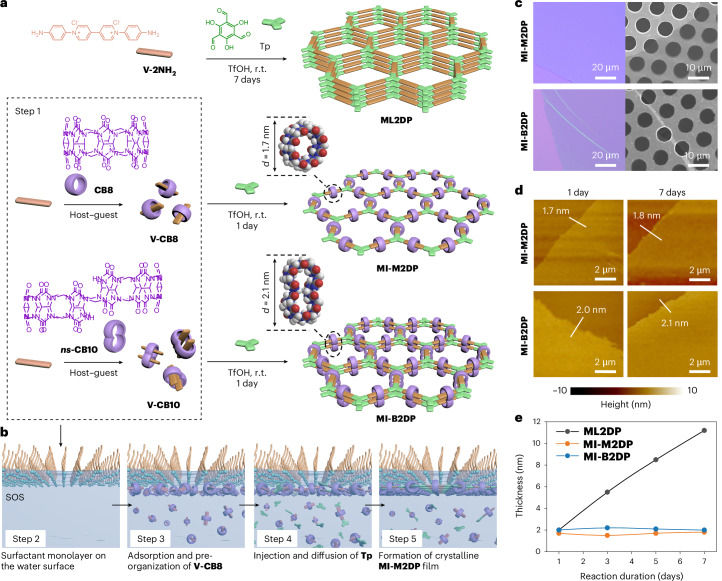
On-water surface synthesis and characterization of MI-M2DP and MI-B2DP. **a**, Reaction schemes illustrating the synthesis of **ML2DP**, **MI-M2DP** and **MI-B2DP** via (A_2_ + B_3_)-type 2D polycondensation, and a host–guest assembly process between **V-2NH**_**2**_ and macrocyclic host (**CB8**, outer diameter, 1.7 nm; ***ns*-CB10**, outer diameter, 2.1 nm) (step 1). r.t., room temperature. **b**, Schematic illustration of the synthetic procedure through the SMAIS method, involving steps 2–5. **c**, Optical microscopy images of **MI-M2DP** and **MI-B2DP** on SiO_2_/Si substrates and SEM images of **MI-M2DP** and **MI-B2DP** on copper grids with a hole area of ∼20 μm^2^. **d**, AFM images of **MI-M2DP** and **MI-B2DP** on SiO_2_/Si substrates with respect to reaction time. The thicknesses of the films along the white line are marked. **e**, Thickness of **ML2DP**, **MI-M2DP** and **MI-B2DP** on SiO_2_/Si substrates with respect to reaction time. Source data

The synthesis of **MI-M2DP** and **MI-B2DP** using a surfactant monolayer-assisted interfacial synthesis (SMAIS)^30,31^ method on the water surface is illustrated in Fig. 1a,b. First, monomers **V-CB8** and **V-CB10** were synthesized in aqueous solutions by incorporating **CB8** and ***ns*-CB10** into the backbone of **V-2NH**_**2**_, respectively, as building blocks for creating **MI-M2DP** and **MI-B2DP** (step 1). The successful formation of **V-CB8** and **V-CB10** was confirmed by UV–visible absorption and ^1^H NMR studies (Supplementary Figs. 5–7)^32^. Then, a monolayer of sodium oleyl sulfate (SOS) was prepared on the water surface (step 2), followed by the injection of 1 ml mixed aqueous solution of trifluoromethanesulfonic acid (TfOH, 7.4 µmol) and **V-CB8** (2.4 µmol; or **V-CB10** for **MI-B2DP** synthesis) into the water subphase (pH ≈ 1.3). The electrostatic interaction between the SOS monolayer and **V-CB8** (or **V-CB10**) drives their adsorption on the water surface within 2 h (step 3; Supplementary Figs. 8–10). Subsequently, 1 ml aqueous solution of 2,4,6-trihydroxybenzene-1,3,5-tricarbaldehyde (**Tp**, 1.6 µmol) was added to the sublayer of the system to initiate the 2D polycondensation via a Schiff-base reaction (step 4). The polymerization was then kept undisturbed at room temperature for 1 day, affording a pale-yellow film with a scalable lateral size (from ∼12.6 to ∼154.1 cm^2^) on the water surface (step 5; Supplementary Figs. 11 and 12). The reaction was extended to 7 days, aiming to monitor thickness evolution and to demonstrate the critical role of MCMs in controlling the layer number of 2DPs.

### Structural characterizations

Attenuated total reflectance Fourier transform infrared (ATR-FTIR) spectroscopy shows that the stretching vibration of N–H (∼3,323 cm^−1^) from **V-CB8** and **V-CB10**, and –CHO (∼1,640 cm^−1^) from compound **Tp** completely disappeared after polycondensation (Supplementary Figs. 13 and 14), suggesting the complete conversion of monomers into 2DPs. Compared to multilayer 2DP without using MCMs (**ML2DP**), the characteristic FTIR peaks of –CH_2_– (2,945 cm^−1^) and C=O (1,716 cm^−1^) from **V-CB8** and **V-CB10** monomers can be observed in **MI-M2DP** and **MI-B2DP**, supporting the successful embedding of **CB8** and ***ns*-CB10** into the 2DP networks. The model reaction results also reveal that the MCMs remain firmly integrated into the viologen moieties even after the on-water surface reaction (Supplementary Figs. 15–17). Furthermore, the chemical structure and composition of **MI-M2DP** and **MI-B2DP** were confirmed by surface-enhanced Raman and X-ray photoelectron spectroscopy (XPS) characterizations (Supplementary Figs. 18–22 and Supplementary Table 1). Energy dispersive X-ray (EDX) mapping also reveals a homogeneous distribution of carbon, nitrogen, oxygen and fluorine in both 2DP films (Supplementary Figs. 23 and 24).

Optical microscopy and scanning electron microscopy (SEM) images show the macroscopically homogeneous nature of **MI-M2DP** and **MI-B2DP** films (Supplementary Fig. 25). As shown in Fig. 1c and Supplementary Figs. 26 and 27, both **MI-M2DP** and **MI-B2DP** films can suspend over the holes (lateral size, ∼20 μm^2^) on a transmission electron microscopy (TEM) grid without rupturing, indicative of their excellent mechanical stability^33^. AFM analysis of **MI-M2DP** and **MI-B2DP** films shows a root mean square roughness of 0.18 nm and 0.27 nm in an area of 10 × 10 µm^2^ (Supplementary Fig. 28). The thicknesses of **MI-M2DP** and **MI-B2DP** are determined to be ∼1.7 and 2.1 nm, respectively, aligning with the anticipated values for the monolayer and bilayer structures (Fig. 1d and Supplementary Figs. 29 and 30)^34^. In contrast to the observed increase in thickness over time for **ML2DP** (from 2.0 nm after 1 day to 11.2 nm after 7 days), the thicknesses of **MI-M2DP** and **MI-B2DP** are maintained (Fig. 1e and Supplementary Figs. 31–36). These results suggest that the bulky MCMs with single or dual cavities prevent *π*–*π* stacking in 2DPs, enabling precise control over the layer numbers from monolayer to bilayers.

**Fig. 2:**
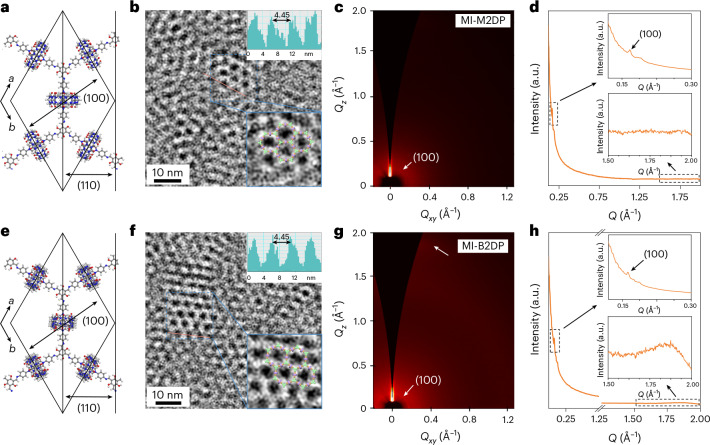
Structural characterizations of MI-M2DP and MI-B2DP films. **a**, Schematic illustration of the **MI-M2DP** unit cell. The (100) and (110) planes of **MI-M2DP** are marked with black arrows. **b**, HRTEM image of **MI-M2DP**. Top inset: intensity profiles along the red line. Bottom inset: magnified HRTEM images with the honeycomb structure overlaid. **c**,**d**, GIWAXS pattern (**c**) and the integrated intensity profile (**d**) of **MI-M2DP** film. *Q* represents the integrated scattering vector of in-plane (*Q*_*xy*_) and out-of-plane (*Q*_*z*_). **e**, Schematic illustration of the **MI-B2DP** unit cell. The (100) and (110) planes of **MI-B2DP** are marked with black arrows. **f**, HRTEM image of **MI-B2DP**. Top inset: intensity profiles along the red line. Bottom inset: magnified HRTEM images with the honeycomb structure overlaid. **g**,**h**, GIWAXS pattern (**g**) and the integrated intensity profile (**h**) of **MI-B2DP** film. Source data

To date, characterizing the crystal structure of monolayer and bilayer 2DPs through TEM and synchrotron-based grazing-incidence wide-angle X-ray scattering (GIWAXS) remains challenging due to their sensitivity to high-energy radiation. To mitigate the structural degradation caused by electron-radiation-induced knock-on damage, electrostatic charging and chemical etching^35^, we lowered the voltage and electron dose rate of TEM, and applied a graphene encapsulation method to enhance their radiation resistance (**G/MI-M2DP/G** and **G/MI-B2DP/G**), as shown in Supplementary Figs. 37–43. High-resolution TEM (HRTEM) resolves the hexagonal structure of **MI-M2DP** with a lattice parameter of *a* = *b* = 44.5 Å, *γ* = 120° (Fig. 2a,b and Supplementary Fig. 44). The selected-area electron diffraction (SAED) pattern of **G/MI-M2DP/G** shows a weak diffraction ring at 0.45 nm^−1^ (*d* spacing, 22.2 Å) attributed to the (110) crystal plane (Supplementary Fig. 40), indicating a polycrystalline nature. To probe the macroscopic structural order of **MI-M2DP**, we further performed GIWAXS measurement on a 20-layer **MI-M2DP** film prepared through layer-by-layer (LBL) assembly. The in-plane reflection ring at *Q*_*xy*_ = 0.17 Å^−1^ (that is, *d* spacing, 37.0 Å) agrees well with the (100) plane of **MI-M2DP** (Fig. 2c,d and Supplementary Fig. 43), confirming its in-plane crystal structure.

The same characterizations were then carried out for **MI-B2DP** samples (Fig. 2e). The hexagonal lattice of **MI-B2DP** (*a* = *b* = 44.5 Å, *γ* = 120°) was observed in HRTEM images (Fig. 2f and Supplementary Fig. 44). The SAED pattern shows an obvious reflection at 0.45 nm^−1^ (*d* spacing, 22.2 Å), which can be assigned to the (110) plane of **MI-B2DP** (Supplementary Fig. 41). The GIWAXS pattern displays a diffraction ring at *Q*_*xy*_ = 0.17 Å^−1^ (that is, *d* spacing, 37.0 Å), corresponding to the (100) in-plane parameter of **MI-B2DP**. Additionally, a weak reflection peak at *Q*_*z*_ = 1.86 Å^−1^ suggests an interlayer distance of 3.4 Å between **MI-B2DP** bilayers (Fig. 2g,h and Supplementary Fig. 43)^17^. These results validate the successful synthesis of polycrystalline **MI-M2DP** and **MI-B2DP** films, lending further credence to the feasibility of utilizing MCMs for modulating the out-of-plane structure of 2DPs.

### SIEBIMM measurements

**Fig. 3:**
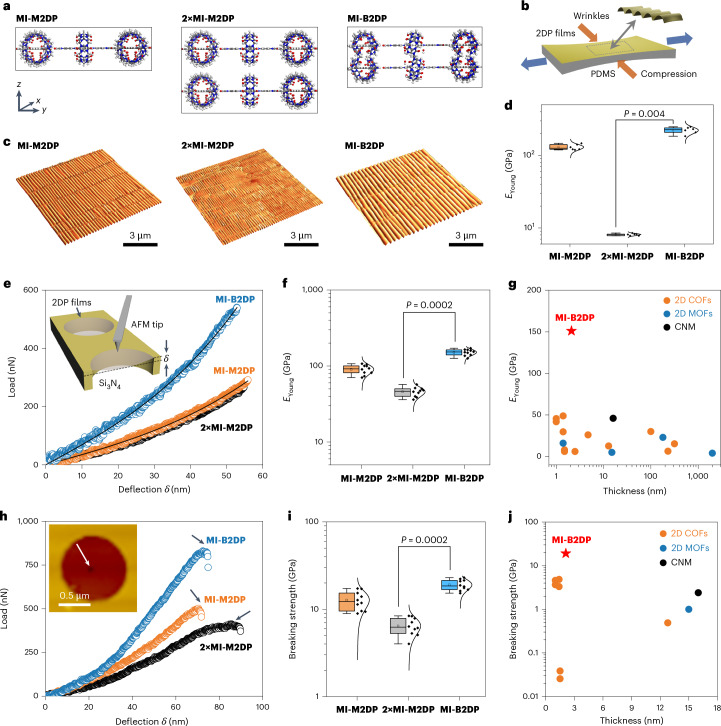
Mechanical properties of MI-M2DP, 2×MI-M2DP and MI-B2DP. **a**, Side view of **MI-M2DP**, **2×MI-M2DP** and **MI-B2DP**. **b**, Schematic illustration of the lateral compression of PDMS during uniaxial transverse stretching, inducing monolayer wrinkling. **c**, AFM topographical images of the large-scaled wrinkle pattern (10 × 10 µm^2^) of **MI-M2DP**, **2×MI-M2DP** and **MI-B2DP** at 10% compressive strain. **d**, *E*_Young_ of **MI-M2DP**, **2×MI-M2DP** and **MI-B2DP**. All values are expressed as mean ± s.d., *n* = 6, *P* = 0.004. **e**, Load–deflection and fitting curves of **MI-M2DP**, **2×MI-M2DP** and **MI-B2DP** films. Inset: schematic diagram of the AFM nanoindentation measurements. **f**, *E*_Young_ of **MI-M2DP**, **2×MI-M2DP** and **MI-B2DP** films. All values are expressed as mean ± s.d., *n* = 10, *P* = 0.0002. **g**, Young’s modulus comparison of **MI-B2DP** with the reported layer-stacked 2D COFs, 2D MOFs films and CNM measured by AFM nanoindentation. **h**, Load–deflection curves (up to fracture) of **MI-M2DP**, **2×MI-M2DP** and **MI-B2DP** films. Inset: AFM image of the fractured 2DP film suspended over the hole after the indentation. **i**, Breaking strengths of **MI-M2DP**, **2×MI-M2DP** and **MI-B2DP** films. All values are expressed as mean ± s.d., *n* = 10, *P* = 0.0002. **j**, Breaking strength comparison of **MI-B2DP** with the reported layer-stacked 2D COFs, 2D MOFs films and CNM measured by AFM nanoindentation. In the box plots, the centre line is the median, box limits are the first and third quartiles, and whiskers are 1.5× the interquartile range. Significant difference *P* values were determined by two-sided Mann–Whitney tests. Source data

To determine the mechanical properties of **MI-M2DP** and **MI-B2DP**, the SIEBIMM technique was initially used^22^. The synthesized 2DP films were horizontally transferred onto a polydimethylsiloxane (PDMS) elastomeric support and strained using a motorized strain device, resulting in the formation of a regular wrinkling pattern perpendicular to the strain direction in 2DPs (Supplementary Fig. 45). Three samples, namely, **MI-M2DP**, two-layer stacked **MI-M2DP** (**2×MI-M2DP**) and **MI-B2DP**, were measured to investigate the impact of interlayer interactions on the mechanical properties (Fig. 3a,b). AFM topographical images show regular wrinkle patterns for all samples (Fig. 3c), indicating their high quality and suitability for SIEBIMM. The wavelengths of the wrinkles in **MI-M2DP**, **2×MI-M2DP** and **MI-B2DP**, calculated by a Python-based calculation method and cross-checked by 2D Fourier-transformation^36^, were 274 ± 10, 188 ± 7 and 402 ± 31 nm, respectively (Supplementary Figs. 46 and 47). The *E*_Young_ was thus evaluated using the regular wrinkling wavelength, film thicknesses (Supplementary Fig. 48 and Supplementary Table 2) and the mechanical stiffness of the PDMS substrate (2.06 MPa), as described by the following equation^37,38^.

1 $${E}_{\mathrm{Young\_f}}=\frac{3{E}_{\mathrm{Young\_s}}}{\left(1-{{v}_{{\rm{s}}}}^{2}\right)}{\left(\frac{\lambda }{2{\rm{\pi }}h}\right)}^{3}(1-{{v}_{{\rm{f}}}}^{2})$$

where $\lambda$ represents the wrinkle wavelength, *h* is the thickness of the upper film, *ν* is Poisson’s ratio, *E*_Young_f_ is the *E*_Young_ of film and *E*_Young_s_ is the *E*_Young_ of substrate.

The *E*_Young_ values for **MI-M2DP**, **2×MI-M2DP** and **MI-B2DP** are 130 ± 27, 8 ± 5 and 222 ± 60 GPa, respectively (Fig. 3d and Supplementary Figs. 49–51). The *E*_Young_ of the mechanically interlocked **MI-B2DP** increases by 71% compared with **MI-M2DP**, while the *E*_Young_ of the vdW-stacked **2×MI-M2DP** decreases by 94%. These results highlight that the mechanical interlocking confers strongly coupled layers to **MI-B2DP**, enhancing the mechanical properties of 2DPs from monolayer to bilayer. Thereby, the resulting *E*_Young_ of **MI-B2DP** is much higher than those of the multilayer polymer films and carbon nanomembranes (CNMs) measured by SIEBIMM methods (Supplementary Fig. 52)^22,39–42^.

### AFM nanoindentation measurements

To verify the mechanical properties of these synthetic films, we further conducted AFM nanoindentation measurements. To this end, the film samples were transferred onto Si_3_N_4_ substrates with 1-μm circular holes (Supplementary Fig. 53). As shown in Fig. 3e, we plotted the non-linear load versus deflection (*F*–*δ*) curves for **MI-M2DP**, **2×MI-M2DP** and **MI-B2DP** films, respectively, which were fitted using a cubic *F*–*δ*^3^ relationship (*R*^2^ > 0.99):

2 $$F=\left({\sigma }_{0}^{2{\rm{D}}}{\rm{\pi }}\right)\delta +\left(\frac{{E}^{2{\rm{D}}}{q}^{3}}{{r}^{2}}\right){\delta }^{3}$$

where *F* is the force applied to the suspended film, *δ* is the deflection of the suspended film, ${\sigma }_{0}^{2{\rm{D}}}$ is the prestrain in units of N m^−1^, *E*^2D^ is the 2D elastic modulus in units of N m^−1^, *r* is the radius of the hole and *q* = 1/(1.05 − 0.15*ν* − 0.16*ν*^2^) is a constant determined by Poisson’s ratio *ν*. The *ν* and *q* values of the measured 2DP films were calculated to be ∼0.3 and ∼0.99, respectively.

From these parameters, we determined the *E*^2D^ values for the **MI-M2DP**, **2×MI-M2DP** and **MI-B2DP** films to be 154 ± 22, 137 ± 19 and 318 ± 30 N m^−1^, respectively. Using the equation *E*_Young_ = *E*^2D^/*h*, where *h* represents the thickness of the samples, their *E*_Young_ were calculated as 90 ± 14, 46 ± 11 and 151 ± 16 GPa, respectively (Fig. 3f). Parallel experiments and simulations demonstrate that the impact of the stacking modes on the *E*_Young_ of **2×MI-M2DP** is negligible (Supplementary Figs. 54 and 55). Compared to **MI-M2DP**, the *E*_Young_ of mechanically interlocked **MI-B2DP** is 67.5% higher, while the *E*_Young_ of vdW-stacked **2×MI-M2DP** decreases by 49.3%, which aligns well with the SIEBIMM results. The *E*_Young_ of **MI-M2DP** is comparable to those of other well-known monolayer 2D materials, such as MoS_2_ (270 GPa)^43^, WS_2_ (272 GPa)^44^, Ti_3_C_2_T_*x*_ (333 GPa)^45^ and graphene (1 TPa)^46^. Furthermore, **MI-B2DP** demonstrates an exceptionally high *E*_Young_ among the reported layer-stacked 2D COFs, 2D metal–organic frameworks (2D MOFs) and CNM (∼2–450 layers; Fig. 3g and Supplementary Table 3) measured by AFM nanoindentation^11,47^.

Next, we attempted to investigate the fracture behaviour upon applying higher loads (Supplementary Figs. 56–60). The films remained suspended on the hole, with nanoscale fracture occurring only in the area of direct contact with the AFM tip. The *F*–*δ* curves of the **MI-M2DP**, **2×MI-M2DP** and **MI-B2DP** films were recorded to determine their fracture loads (*F*_max_; Fig. 3h). The maximum fracture stresses (${\sigma }_{\max }^{2{\rm{D}}}$) of the films were calculated using the formula of the linearly elastic membrane under a spherical indenter:

3 $${\sigma }_{\max }^{2{\rm{D}}}={\left(\frac{{F}_{\max }{E}^{2{\rm{D}}}}{4{\rm{\pi }}{r}_{\mathrm{tip}}}\right)}^{\frac{1}{2}}$$

where *E*^2D^ is the 2D elastic modulus of the suspended film, and *r*_tip_ is the radius of the AFM tip, measured as ∼13.5 nm from the SEM image (Supplementary Fig. 61). The breaking strengths (*σ*) of the **MI-M2DP**, **2×MI-M2DP** and **MI-B2DP** films were determined to be 13 ± 3, 6 ± 2 and 19 ± 3 GPa, respectively, according to the equation *σ* = ${\sigma }_{\max }^{2{\rm{D}}}/h$. (Fig. 3i). Compared to the weak vdW interaction in **2×MI-M2DP**, the mechanical interlocking in **MI-B2DP** tightly integrates the two layers, forming a composite structure that enhances its mechanical properties. The breaking strength of **MI-B2DP** is the highest among layer-stacked 2DP, 2D COF, 2D MOF films, and CNMs (Fig. 3j).

### Theoretical studies of interlayer sliding in bilayer 2DPs

**Fig. 4:**
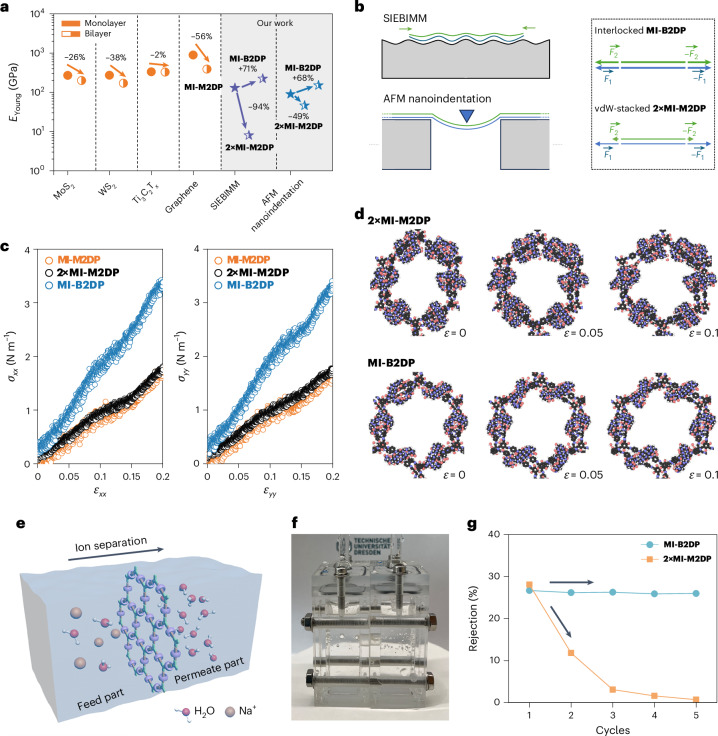
Interlayer behaviour in vdW-stacked 2×MI-M2DP and interlocked MI-B2DP. **a**, Mechanical property comparison of **MI-M2DP**, **MI-B2DP** and **2×MI-M2DP** with those of state-of-the-art monolayer and bilayer 2D materials. **b**, Contributions of the two layers in **2×MI-M2DP** and **MI-B2DP** during SIEBIMM and AFM nanoindentation measurements. **c**, Calculated in-plane stress–strain curves along the zig-zag (*xx*) and armchair (*yy*) directions of **MI-M2DP**, **2×MI-M2DP** and **MI-B2DP**. **d**, Simulated chemical structure of **2×MI-M2DP** and **MI-B2DP** during stress loading. **e**, Schematic illustration of ion separation. Feed part (left), **2×MI-M2DP** or **MI-B2DP** film (middle) and permeate part (right) are marked. **f**, A digital photograph of the ion-separation device. **g**, Na^+^ ion rejection rate of **2×MI-M2DP** and **MI-B2DP** films over five filtration cycles. Source data

Notably, for both SIEBIMM and AFM nanoindentation, the **MI-B2DP** reveals higher *E*_Young_ compared with **MI-M2DP**. This stands as a unique phenomenon because, typically, when stacking monolayers into bilayer 2D materials, interlayer sliding impedes the collective engagement of the layers in the stress–strain process, leading to mechanical relaxation and subsequent degradation of their *E*_Young_ (Fig. 4a)^43–45,48^. However, the mechanically interlocked structure tightly integrates the layers, forming a composite layer that enables simultaneous contributions to the mechanical strength of 2DP films (Fig. 4b). To gain insight into the underlying mechanism of the interlayer sliding in these bilayer systems, we calculated the energy penalty as shown in Supplementary Fig. 62. The sliding energy of **MI-B2DP** is consistently higher (by up to 200 kcal mol^−1^) compared with **2×MI-M2DP**, indicating stronger interlayer coupling in the mechanically interlocked structures. To understand the structural reinforcement in **MI-B2DP** enabled by MCMs, we further performed classical molecular dynamics (MD) simulations to investigate the stress–strain behaviour of **2×MI-M2DP** and **MI-B2DP**. In the simulations, the top layer of **2×MI-M2DP** was weakly coupled to the bottom layer, enabling its free movement during the stress loading. As shown in Fig. 4c,d and Supplementary Figs. 63–67, the interlocked **MI-B2DP** possesses an enhanced in-plane stress response to applied strain, characterized by a higher *E*_Young_ (*E*_Young_ = (216 ± 1) × 10^−2^ GPa) compared with **2×MI-M2DP** (*E*_Young_ = (77 ± 1) × 10^−2 ^GPa), which supports the experimental findings. The weak vdW interaction in **2×MI-M2DP** facilitates the interlayer sliding, thereby reducing the contribution of the chemical bonds in the top layer to the overall stiffness and modulus of the film. In contrast, the synthetic **MI-B2DP** is highly interconnected via MCMs throughout its thickness, which effectively prevents the interlayer sliding. This interconnection ensures that all covalent bonds in the interlocked bilayers and the MCMs simultaneously contribute to the stress response, leading to the reinforced stiffness and modulus of **MI-B2DP**.

### Seawater desalination

To evaluate the practical utility of the enhanced mechanical properties, we assessed the cation separation performance of both **2×MI-M2DP** and **MI-B2DP** films. The as-prepared films were mounted between two reservoirs filled with 0.2 M NaCl solution (feed part) and 2 M sucrose solution (permeate part), in which substantial stress was applied to the films (Fig. 4e,f). After five filtration cycles (72 h per cycle), the Na^+^ rejection rate of **2×MI-M2DP** declined sharply by ∼97.5%, indicative of structural disruption (Fig. 4g). In contrast, **MI-B2DP** maintained a nearly unchanged rejection rate, with only a ∼2.6% decrease over the same period. These results highlight the critical role of mechanical interlocking in preserving structural integrity under prolonged operation, thereby enabling sustained ion-separation performance.

## Discussion

In summary, we have demonstrated the efficient synthesis of crystalline **MI-M2DP** and **MI-B2DP** films on the water surface through the incorporation of MCMs with one and two cavities in their backbones. These embedded bulky MCMs enable the formation of periodic and dense mechanical bonds in 2DPs and facilitate precise control over the number of interlocking layers. TEM and X-ray scattering techniques showed a hexagonal porous structure of **MI-M2DP** and **MI-B2DP** films with an in-plane parameter of *a* = *b* = 44.5 Å. We have shown that the mechanically interlocked structure in **MI-B2DP** suppresses the interlayer sliding and tightly integrates the two layers as a composite layer. Such features enable both layers to contribute to the mechanical strength of 2DP films, leading to **MI-B2DP** having an exceptionally high *E*_Young_ (222 ± 60 GPa from SIEBIMM measurements; 151 ± 16 GPa from AFM nanoindentation), compared with those of **MI-M2DP** (130 ± 27 GPa; 90 ± 14 GPa), vdW-stacked **2×MI-M2DP** (8 ± 5 GPa; 46 ± 11 GPa) and other reported layer-stacked 2D COFs, 2D MOFs and CNM (<50 GPa). Our results contradict the common negative correlation between layer number and *E*_Young_ observed in 2D materials, underscoring the significance of MCMs in reinforcing the structure of 2DPs. As a demonstration of practical utility, we further integrated **MI-B2DP** into a water desalination device that exhibited enhanced durability compared with **2×MI-M2DP** due to its higher mechanical properties. Our findings not only offer insights into the precise control over the synthesis of monolayer and bilayer 2DPs, but also pave the way for reinforcing the mechanical properties of 2D materials from monolayer to multilayer.

## Methods

### Synthesis of MI-M2DP and MI-B2DP films

First, 1 mg of **V-2NH**_**2**_, 3.24 mg of **CB8** (or 1.99 mg of ***ns*-CB10**) and 1 ml of TfOH (7.4 µmol) aqueous solution were added into a glass bottle, and sonicated for 30 min to prepare the **V-CB8** (or **V-CB10**). Then, 50 ml of Milli-Q water was added to a crystallization dish (60 ml; diameter, ∼6 cm), after which 10 μl of SOS solution (1 mg ml^−1^ in chloroform) was spread on the water surface by a micron injector. The chloroform on the water surface evaporated in 30 min. Subsequently, 1 ml of the prepared **V-CB8** (or **V-CB10**) solution was gently injected into the water subphase by using a syringe. After 2 h, 1 ml aqueous solution of **Tp** (1.6 µmol) was injected into the system for 2D polymerization. The reaction was kept undisturbed at room temperature for 1 day. The synthesized **MI-M2DP** (or **MI-B2DP**) film was transferred onto different substrates by a horizontal dipping method, after which it was washed with chloroform, ethanol and Milli-Q water, and dried at 50 °C for 1 h.

### SIEBIMM technique

The PDMS system Sylgard 184 (Dow Corning) was mixed in a 10:1 mass ratio of oligomeric base to curing agent and degassed for 2 min at 2,200 r.p.m. with a ARE250 tumbling mixer (Thinky). The mixture was cast into a poly(styrene) case with a thickness of 2 mm and cured first at room temperature for 48 h followed by a postcuring step at 80 °C for 4 h. The cured PDMS was cut into 45 mm × 10 mm × 2 mm specimens. To activate the surface, a pretreatment step with 10 wt% aqueous HCl solution for 16 h was necessary to provide sufficient adhesion to the 2DPs^49^. These samples were used to lift out different synthesized 2DPs from the water surface to create the necessary bilayer system for wrinkling. To induce the wrinkling, the samples were uniaxially strained to 20% with a motorized strain device, which introduces a perpendicular compressive strain with a Poisson ratio of 10%. The resulting wrinkle pattern was analysed with an AFM (FastScan, Bruker), which was operated in tapping mode with NanoScope 9.3 software. A Tap300 cantilever (tip radius, 15 nm; spring constant, 40 N m^−1^; nominal resonance frequency, 300 kHz) was used for topographical measurements. The images were taken at sizes of 50 × 50 mm^2^ and 10 × 10 mm^2^ using a resolution of 512 × 512 pixels. The wrinkle wavelength was determined using a Python-based (Python 3.0) wrinkle calculation script based on the topographical images^36^.

### AFM nanoindentation measurement

The AFM nanoindentation measurement was performed using a Park Systems NX10. Single-crystal diamond tips (AD-40-SS) were utilized for the measurement. Before the nanoindentation, the calibration of the deflection sensitivity (converting the voltage measured by the photodetector into the deflection in nanometres) was performed on a sapphire substrate. Then, the spring constant was corrected to 48.284 N m^−1^ by using the Sader method^50^. During the measurements, the indentation speed was controlled at 0.05 μm s^−1^, and the *F*–*δ* curves were recorded to evaluate the mechanical property of the 2DPs.

### Simulations of elastic properties

The elastic properties were simulated using classical MD simulations with the Large-scale Atomic/Molecular Massively Parallel Simulator (LAMMPS)^51^ with a parametrization of the force-field Reax-FF^52^ at 10 K. All simulations were performed with a time-step of 0.25 fs. First, a monolayer of the 2DP (without **CB8** or **CB10**) was geometry relaxed and equilibrated using the Nosé–Hoover barostat without external pressure with a temperature damping of 25 fs and a stress damping of 250 fs in a NpT ensemble for 400,000 time-steps. Based on the cell size of the last time-step of this simulation, all other systems were scaled for all further calculations. Next, the respective system was equilibrated in a NVT ensemble for another 100,000 time-steps. The equilibrated system was stretched along the *x* or *y* direction within two different strain ranges. A short strain range yields a maximum strain of 4%, where the system was strained with a strain rate of 0.000001 fs^−1^ for 160,000 time-steps. The stresses were recorded during the simulation and the strain components were calculated based on the relative change of the box length and width. The elements of the stiffness tensor were calculated to evaluate their 2D bulk, shear and *E*_Young_ (ref. ^53^). To investigate the rupture behaviour, a large strain range with a strain rate of 0.000005 fs^−1^ for 300,000 time-steps was used, yielding a maximum strain of 37.5%. For the simulation of the interlayer shearing, first a NVT equilibration was performed for 100,000 time-steps. Then, several carbon atoms at the top and at the bottom layer were fixed along the *x* direction and another short NVT equilibration of 10,000 time-steps was performed. The fixed atoms of the two layers were subsequently shifted against each other along *x* with a velocity of 7.46 × 10^−4^ Å fs^−1^ and −7.46 × 10^−4^ Å fs^−1^, respectively, over 100,000 time-steps. During the interlayer shearing, the total energy of the system was recorded.

## Supplementary information

Supplementary Information Supplementary Figs. 1–67, discussion and Tables 1–3.

## Source data

Source Data Fig. 1 Thickness with respect to reaction time.

Source Data Fig. 2 GIWAXS integrated intensity profile.

Source Data Fig. 3 Mechanical properties.

Source Data Fig. 4 Calculated mechanical properties and ion separation.

## Data Availability

The data supporting the findings of this study are available within the Article and its Supplementary Information. Experimental procedures and characterizations are available in the Supplementary Information.
