# Supplementary material for: Mechanically interlocked monolayer and bilayer two-dimensional polymers with high elastic modulus
Source: Nat Synth. 2025 Nov 13;5(3):357–66. doi: 10.1038/s44160-025-00930-4 (PMC12987722; doi:10.1038/s44160-025-00930-4)
Supplement: Supplementary file 1 — Supplementary Figs. 1–67, discussion and Tables 1–3. [file 44160_2025_930_MOESM1_ESM.pdf]

nature synthesis

Supplementary information

https://doi.org/10.1038/s44160-025-00930-4

Mechanically interlocked monolayer and bilayer two-dimensional polymers with high elastic modulus

In the format provided by the authors and unedited

Table of Contents

Materials 3
Methods 3
Characterizations 3
Supplementary Fig. 1 5
Supplementary Fig. 2 6
Supplementary Fig. 3 7
Supplementary Fig. 4 8
Supplementary Fig. 5 9
Supplementary Fig. 6 10
Supplementary Fig. 7 11
Supplementary Fig. 8 12
Supplementary Fig. 9 13
Supplementary Fig. 10 14
Supplementary Fig. 11 15
Supplementary Fig. 12 16
Supplementary Fig. 13 17
Supplementary Fig. 14 18
Supplementary Fig. 15 19
Supplementary Fig. 16 20
Supplementary Fig. 17 21
Supplementary Fig. 18 22
Supplementary Fig. 19 23
Supplementary Fig. 20 24
Supplementary Fig. 21 25
Supplementary Fig. 22 26
Supplementary Fig. 23 27
Supplementary Fig. 24 28
Supplementary Fig. 25 29
Supplementary Fig. 26 30
Supplementary Fig. 27 31
Supplementary Fig. 28 32
Supplementary Fig. 29 33
Supplementary Fig. 30 34
Supplementary Fig. 31 35
Supplementary Fig. 32 36
Supplementary Fig. 33 37
Supplementary Fig. 34 38
Supplementary Fig. 35 39
Supplementary Fig. 36 40
Supplementary Fig. 37 41
Supplementary Fig. 38 42
Supplementary Fig. 39 43

Supplementary Fig. 40 44
Supplementary Fig. 41 45
Supplementary Fig. 42 46
Supplementary Fig. 43 47
Supplementary Fig. 44 48
Supplementary Fig. 45 49
Supplementary Fig. 46 50
Supplementary Fig. 47 51
Supplementary Fig. 48 52
Supplementary Fig. 49 53
Supplementary Fig. 50 54
Supplementary Fig. 51 55
Supplementary Fig. 52 56
Supplementary Fig. 53 57
Supplementary Fig. 54 58
Supplementary Fig. 55 59
Supplementary Fig. 56 60
Supplementary Fig. 57 61
Supplementary Fig. 58 62
Supplementary Fig. 59 63
Supplementary Fig. 60 64
Supplementary Fig. 61 65
Supplementary Fig. 62 66
Supplementary Fig. 63 67
Supplementary Fig. 64 68
Supplementary Fig. 65 69
Supplementary Fig. 66 70
Supplementary Fig. 67 71
Supplementary Table 1 72
Supplementary Table 2 73
Supplementary Table 3 74
References 75

Supplementary experimental sections

Materials

1,1′-bis(4-aminophenyl)-[4,4′-bipyridine]-1,1′-diium chloride (V-2NH_2) and 2,4,6-trihydroxybenzene-1,3,5-tricarbaldehyde (Tp) were purchased from BLD Pharm Deutschland, GmbH (Germany). Sodium oleyl sulfate (SOS), glycoluril and paraformaldehyde were obtained from Sigma- Aldrich, GmbH (Germany). Cucurbit[8]uril (**CB8**) was purchased from Ambeed Deutschland, GmbH (Germany). Monolayer graphene was purchased from Graphenea Deutschland, GmbH (Germany). All compounds were used as received.

Silicon substrates (1 cm×1 cm) for scanning electron microscopy (SEM). SiO_2 (300 nm)/Si substrates (1 cm×1 cm) were used for optical microscopy (OM) and atomic force microscopy (AFM) measurements. Gold-coated silicon wafer from Sigma-Aldrich was used for Fourier-transform infrared spectroscopy (FTIR), Raman spectroscopy, and X-ray photoelectron spectroscopy (XPS) tests. Quartz plate from Plano GmbH, Germany, (1.5 cm×1.5 cm, 1 mm thickness) was used for UV-Vis absorption spectroscopy investigations. Copper meshes from Plano GmbH were used for the TEM measurements.

Substrates cleaning

The cleanness of substrates is crucial in surface science. The surface of the Silicon substrates, SiO_2(300 nm)/Si substrates, and Quartz plates substrates were cleaned with a fresh piranha solution (80 vol.% H_2SO_4:20 vol.% H_2O_2 30 % aqueous solution) for 1 h. Then, 100 ml Milli-Q water and 100 ml isopropanol was utilized to wash the surface under sonication, respectively.

Methods

Synthesis of ns-CB10. Synthesis of ***ns*-CB10** was successfully reproduced by following the literature-reported procedure,[1] and the performed synthetic procedure is as follows. Glycoluril (1.42 g, 9.99 mmol) and paraformaldehyde (0.50 g, 16.69 mmol) were added to conc. HCl (4 ml), and heated at 50 ºC for 3 days. The obtained precipitate was separated by centrifugation to yield a crude solid (~400 mg). The crude solid was washed with HCl:H_2O (1:1, v/v), 0.2 M Na_2SO_4, and finally H_2O to yield *ns*-CB10 (312 mg, 0.19 mmol) as a white solid. ^{1}H NMR (300 MHz, D_2O): 5.65-5.45 (s, 36H), 4.77 (s, 4H), 4.19 (m, 16H).

Characterizations

^{1}H NMR spectra were performed at 30 ºC on a Bruker AV-II 300 spectrometer, and the ASAP-Mass spectrum was obtained via the machine expression CMS. FT-IR spectra were recorded by using a Bruker Optics ALPHA-E spectrometer equipped with Attenuated Total Reflectance (ATR) module. A Cary 5000 UV-vis-near infrared (NIR) Spectrophotometer was used to test the adsorption spectra. XPS spectra were obtained by using an X-ray photoelectron spectroscopy (XPS, XSAM800, Kratos Analytical, UK). Optical microscopy (Zeiss) and AFM (NT-MDT) were used to measure the morphology and thickness of 2DPs. TEM was conducted using Libra 200 kV (Zeiss) and JEOL JEM F200 operated at 200 kV acceleration voltage equipped with a GATAN OneView CMOS camera for fast imaging. SEM (Zeiss Gemini 500) equipped with energy-dispersive X-ray spectroscopy was employed to monitor the morphology.

Computational setup for emulating nanoindentation experiments. To investigate the influence of

weak and strong coupling in 2DPs, we consider two different scenarios regarding the coupling strength. First, for strong coupling, both layers experience the same in-plane strain, which is simulated by applying strain to the unit cell of the polymer bilayer. Second, for weak coupling, the bottom layer experiences full strain, while the top layer experiences only the strain that is indirectly induced on it through the strain of the bottom layer. To achieve this computationally, the periodicity of the top layer must be broken and free to move.

UV-vis measurement of host-guest assembly in Step 1. 1 mg of **V-2NH$_2$** and 3.24 mg of **CB8** were dissolved in 1 ml of Milli-Q water, and sonicated for 30 min at room temperature. Then, the as-prepared solution was added to the quartz cuvette for the UV-vis measurements.

^{1}H-NMR measurements of host-guest assembly in Step 1. Five different D_2O solutions of **V-2NH$_2$/CB8** (1 ml) were prepared, containing 1 mg of **V-2NH$_2$** and varying amounts of **CB8** (3.24, 2.43, 1.62, 0.81, 0 mg). These D_2O solutions were sonicated for 30 min at room temperature and then transferred into the standard NMR tubes for the ^{1}H-NMR measurements.

UV-vis measurement of monomer adsorption from Step 3 to Step 5. To monitor the on-water surface reaction, we further performed UV-vis measurements from Step 3 to Step 5. For this purpose, 7.4 μmol of TfOH was added to the obtained **V-CB8** aqueous solution to protonate the **V-CB8** after the host-guest assembly process. Subsequently, the adsorption process occurred upon injecting the V-CB8 solution into the water phase (Step 3). After 2 hours, 1 ml aqueous solution of 2,4,6-trihydroxybenzene-1,3,5-tricarbaldehyde (Tp, 1.6 μmol) was injected into the system for the polymerization (Step 4 and Step 5). The compounds on the water surface were transferred by using quartz plate (1.5 cm×1.5 cm, 1 mm thickness) in different time periods. After drying at 50 °C, the UV-vis absorption spectroscopy was employed to obtain the time-evolved spectra.

Supplementary Figures and Tables

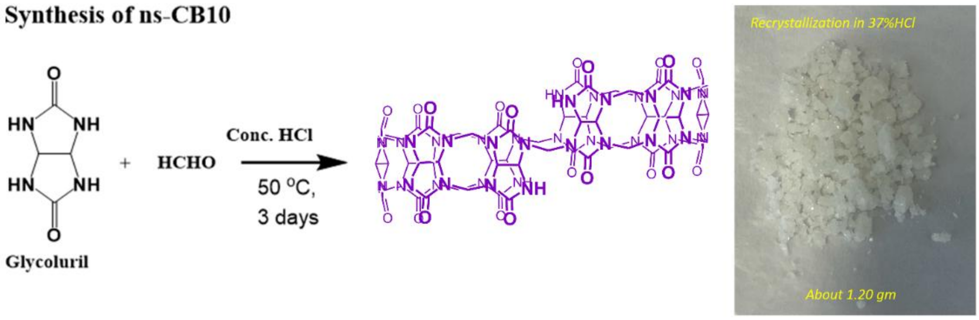

Supplementary Fig. 1. Schematic of the synthesis and the digital photo of *ns*-**CB10**.

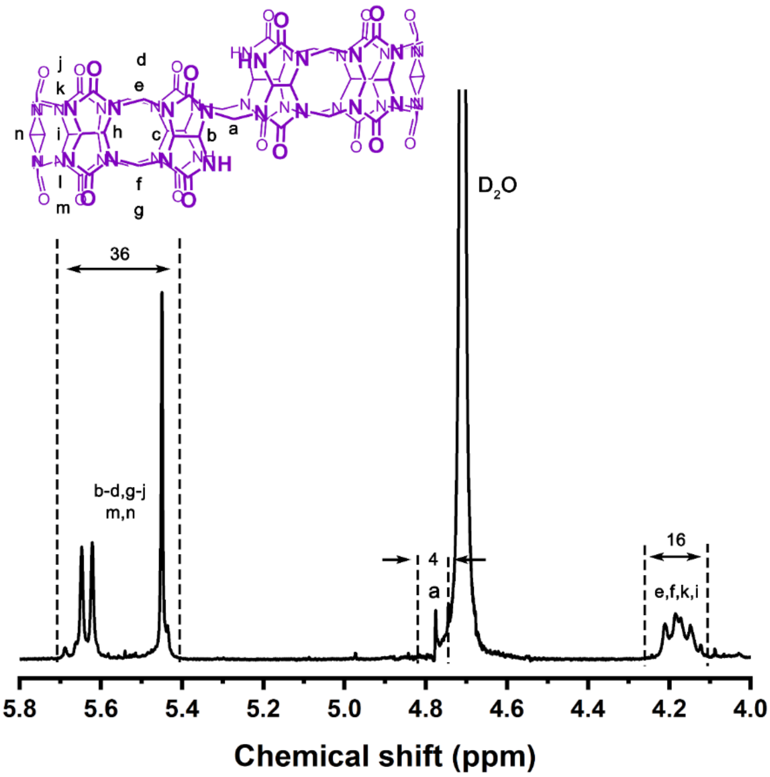

Supplementary Fig. 2. ^{1}H NMR spectrum of *ns*-**CB10** dissolved in D_2O.

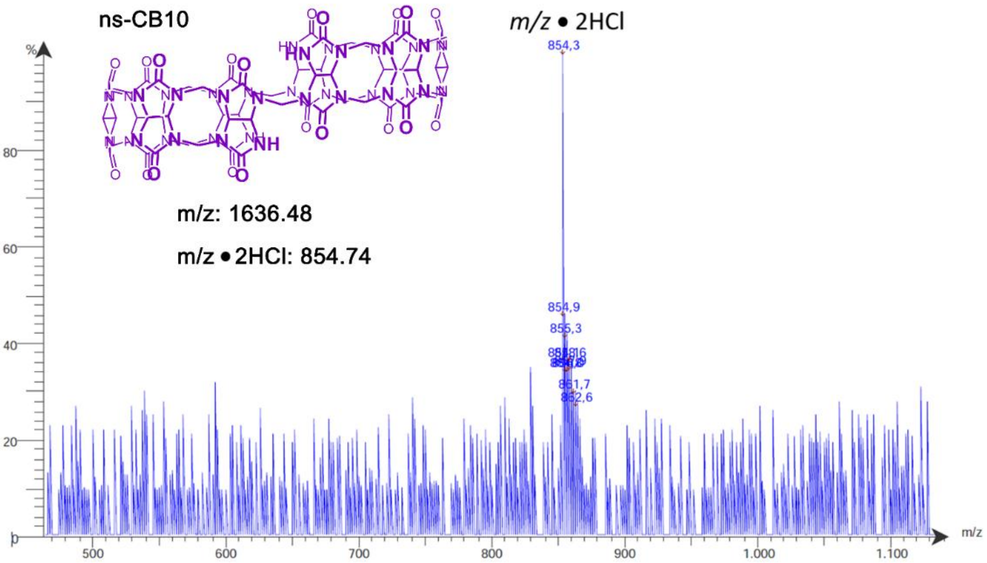

Supplementary Fig. 3. ASAP Mass spectrum of *ns*-**CB10**.

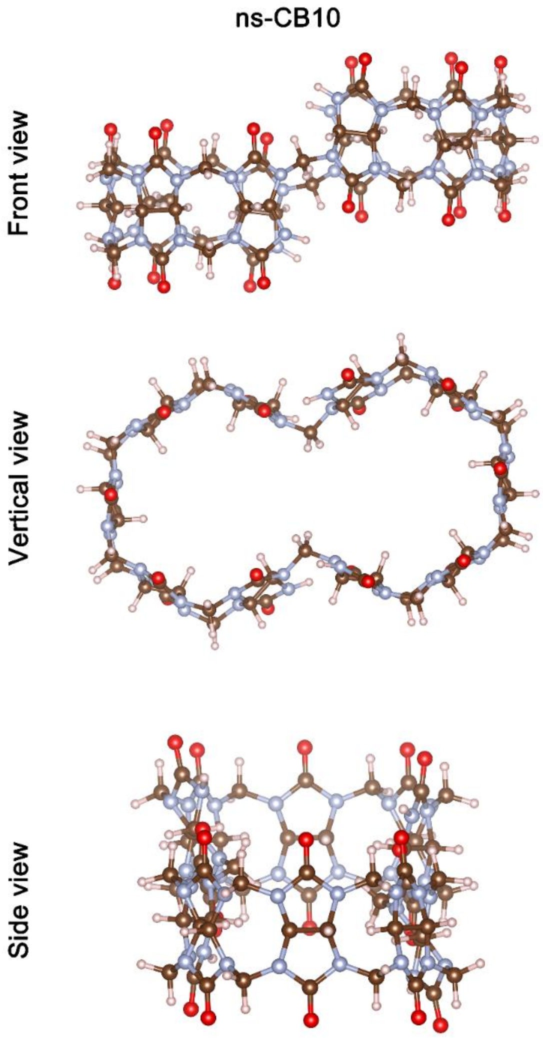

Supplementary Fig. 4. Front, vertical and side views of the calculated *ns*-**CB10** molecule. A density functional geometry optimizations of *ns*-**CB10** were performed with Gaussian16 software package with Becke's three-parameter hybrid exchange functional and the Lee-Yang-Parr correlation functional (B3LYP) and the 6-311G basis set.[2] The distance of optimized *ns*-**CB10** was measured using Materials Studio 8.0.

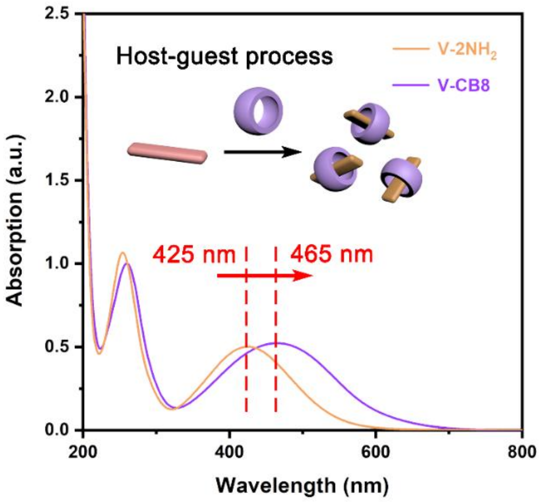

Supplementary Fig. 5. UV-vis spectra of **V-2NH$_2$** (before embedding **CB8**) and **V-CB8** (after embedding **CB8**).

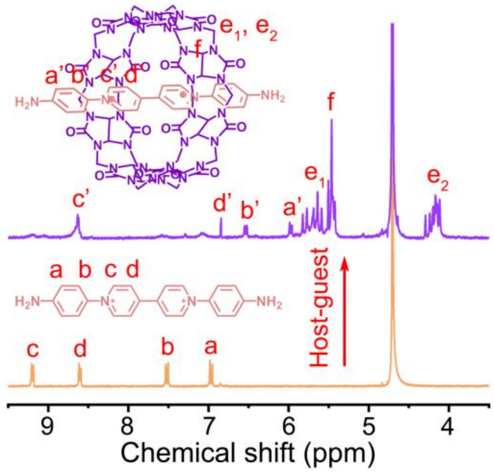

Supplementary Fig. 6. ^{1}H NMR analysis of **V-2NH$_2$** and **V-CB8**.

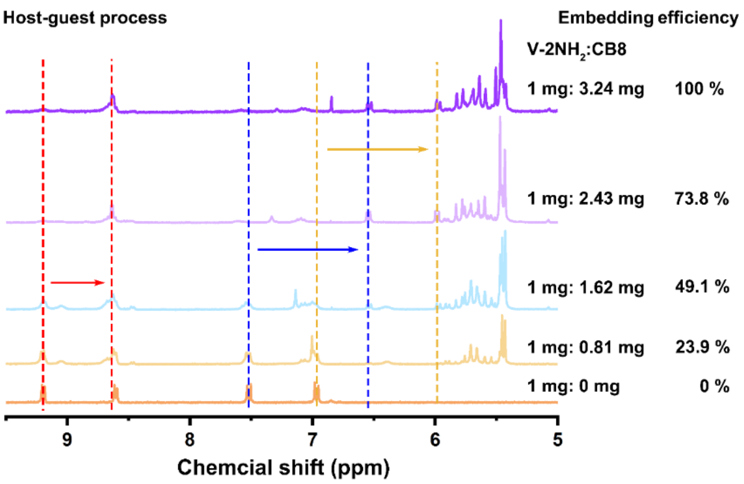

Supplementary Fig. 7. ^{1}H NMR spectra of **V-2NH$_2$/CB8** solutions with different ratios. The embedding efficiency increases from 0 % to 100 %.

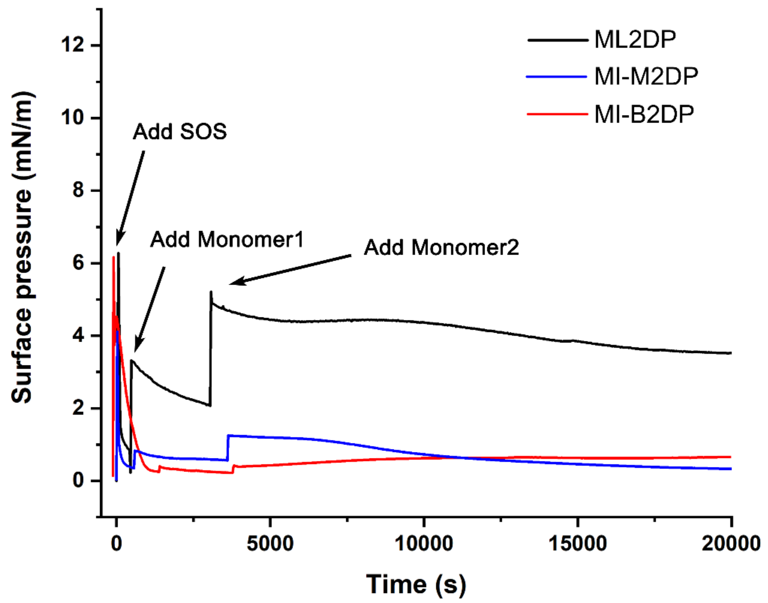

Supplementary Fig. 8. Surface pressure of **ML2DP**, **MI-M2DP** and **MI-B2DP** during the operations. The first three stages in time-dependent surface pressure curves are caused by adding SOS, **V-CB8** and Tp, respectively.

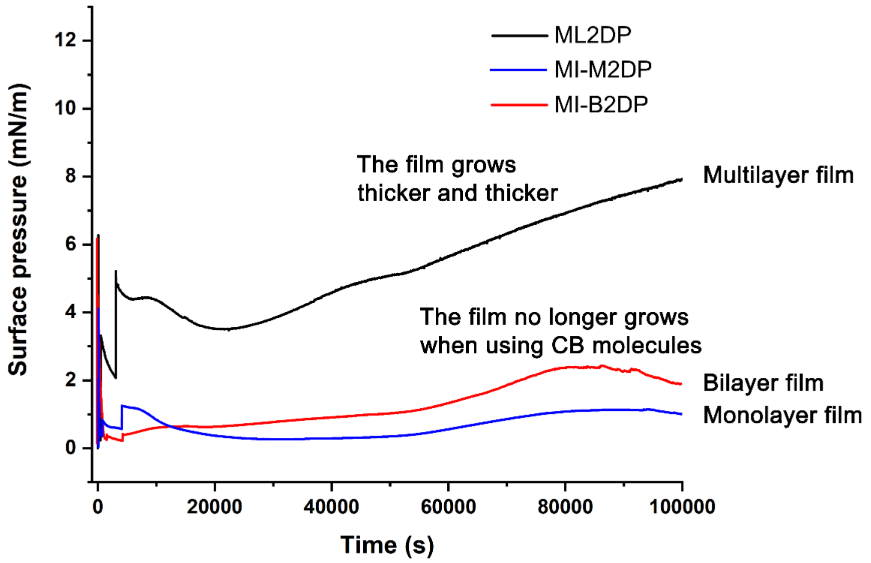

Supplementary Fig. 9. Surface pressure of **ML2DP**, **MI-M2DP** and **MI-B2DP** with respect to reaction time. After triggering the reaction, the surface pressure of **ML2DP** gradually increases, illustrating the continuous growth of the 2DP film. However, for **MI-M2DP**, the film no longer grows thicker after 24h, owing to the embedded **CB8** in the framework which can prevent the π-π stacking between different layers.

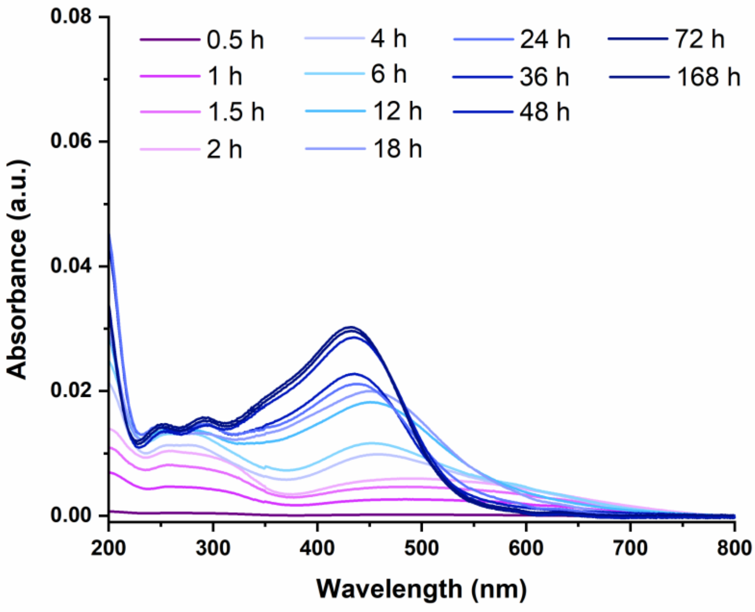

Supplementary Fig. 10. UV-vis spectra of **MI-M2DP** with respect to reaction time. The increasing intensity of the red curves in time-dependent UV-vis spectra reveals the pre-organization process of the **V-CB8**, while a new absorption peak at 428 nm (blue curves) gradually appears indicating the polycondensation process on the water surface.

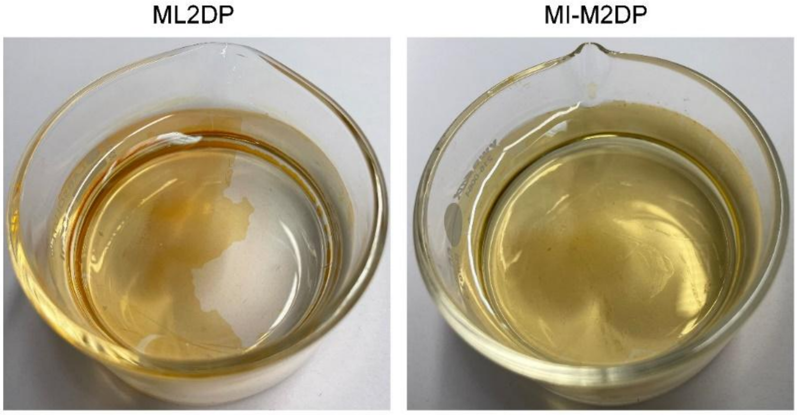

Supplementary Fig. 11. Digital photos of **ML2DP** and **MI-M2DP**.

To evaluate the scalability of this method for large-scale production of the representative MI-M2DP, we conducted additional experiments by increasing the reaction area and refining the synthetic conditions. After 24 h of reaction, we successfully prepared MI-M2DP films with the lateral size of ~12.6, ~28.3, ~50.3, and ~154.1 cm^2 on the water surface as shown in Supplementary Fig. 12. These results reveal that the synthetic method employed in this study offers remarkable scalability and practicality for the production of large-area 2DP films.

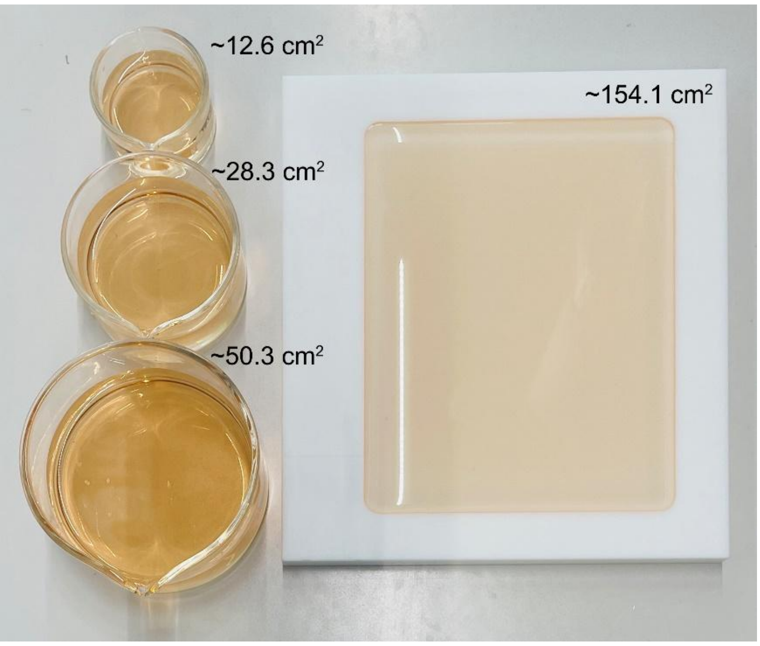

Supplementary Fig. 12. A digital photo of the reaction systems for the scalable synthesis of **MI-M2DP** using the SMAIS method.

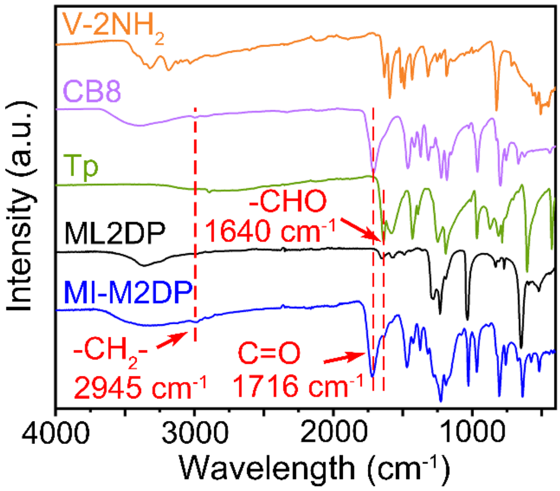

Supplementary Fig. 13. ATR-FTIR spectra of **V-2NH$_2$**, **CB8**, Tp, **ML2DP** and **MI-M2DP** with marked functional groups.

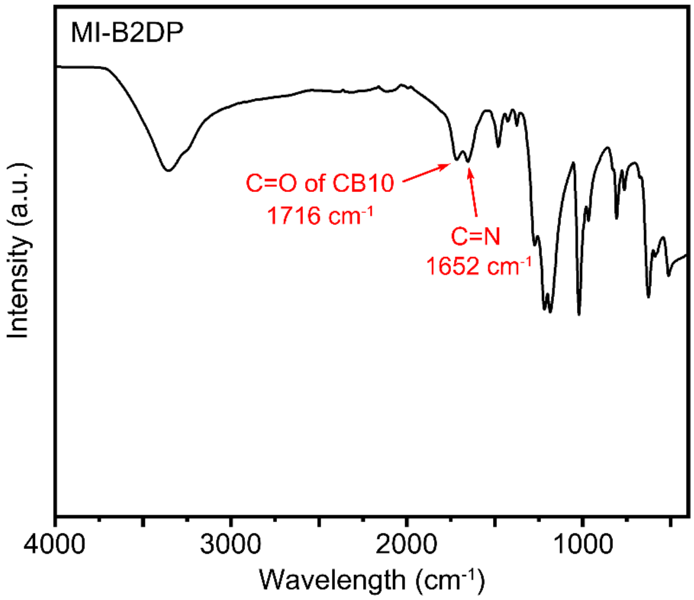

Supplementary Fig. 14. ATR-FTIR spectrum of **MI-B2DP**.

The chemical composition of the final product, particularly the relative ratio of the successfully interlocked macrocyclic molecules (**MCMs**), is indeed a critical aspect of our study. To address this, we attempted to collect the as-synthesized 2DP films on the water surface and re-dissolve them with HCl solution for ^{1}H-NMR measurements. However, the total mass of the synthetic monolayer film (less than 0.001 mg) is significantly below the required minimal amount (>1 mg) for the standard ^{1}H-NMR measurements. Therefore, we were unable to obtain meaningful signals from the NMR analysis.

Instead, we simplified the 2D polymerization and conducted the model reaction using **V-CB8** (**M1**) and 2,4,6-trihydroxybenzaldehyde (**M2**) on the water surface under identical conditions (Supplemental Fig. 15). Due to the π-π interaction and hydrogen bond of their terminal phloroglucinol groups, the model products aggregated on the water surface to form a thick film (~58 nm) after 24 h of reaction (Supplemental Fig. 16). Then, the synthetic films were collected from the on-water surface reaction systems and dissolved in D_2O for ^{1}H-NMR measurements. The chemical shifts of viologen moieties and MCM were observed simultaneously as shown in the ^{1}H-NMR spectrum, and their intensities indicated a molecular ratio of around 1:1 (Supplemental Fig. 17). These results demonstrate that the MCMs remain firmly integrated into the viologen moieties even after the on-water surface reaction.

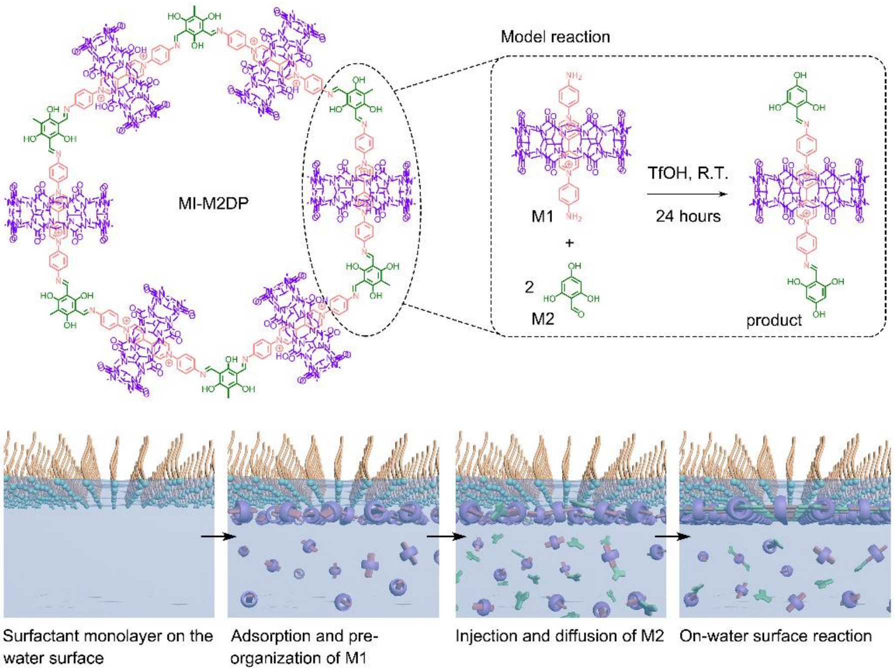

Supplementary Fig. 15. Schematic illustration of the model reaction through on-water surface synthesis.

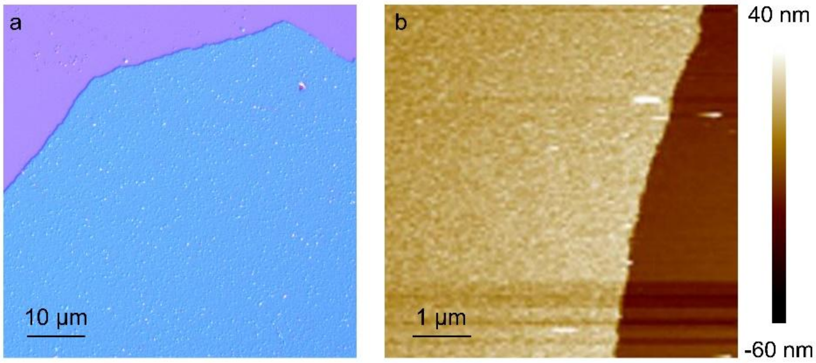

Supplementary Fig. 16. Morphology of the model product film. a,**b**, OM (**a**) and AFM (**b**) images of the model products on SiO_2/Si substrates.

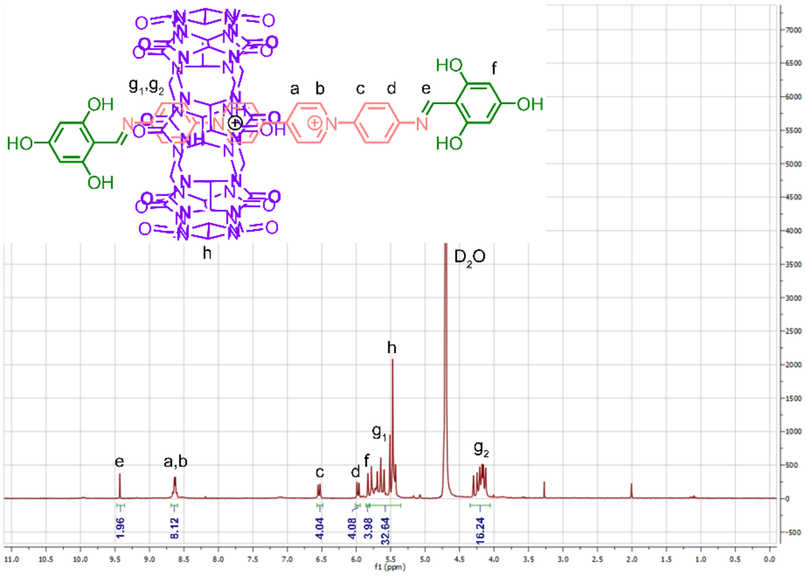

Supplementary Fig. 17. ^{1}H-NMR spectrum of the model reaction product. Inset: the chemical structure of the predicted product.

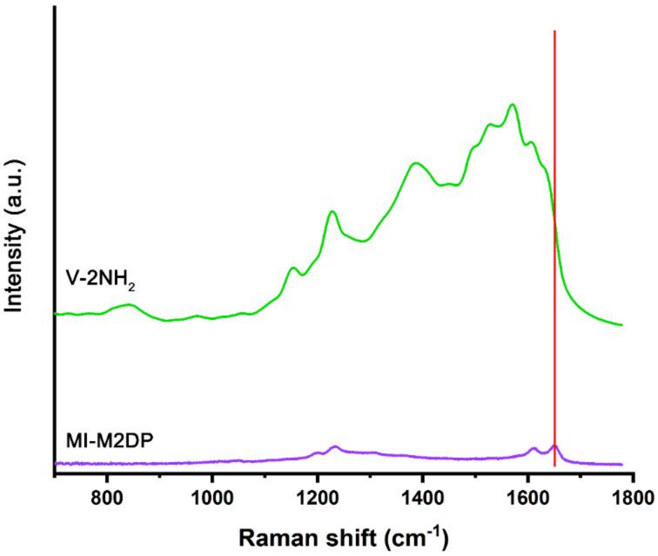

Supplementary Fig. 18. Surface-enhanced Raman spectra of **V-2NH$_2$** and **MI-M2DP**.

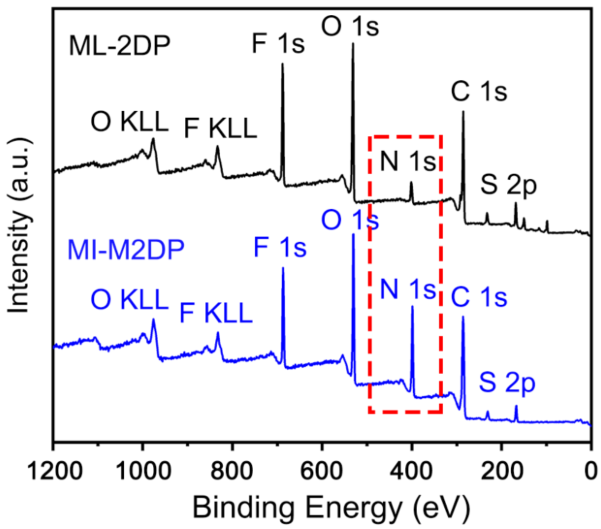

Supplementary Fig. 19. XPS survey scan spectra of **ML2DP** and **MI-M2DP**.

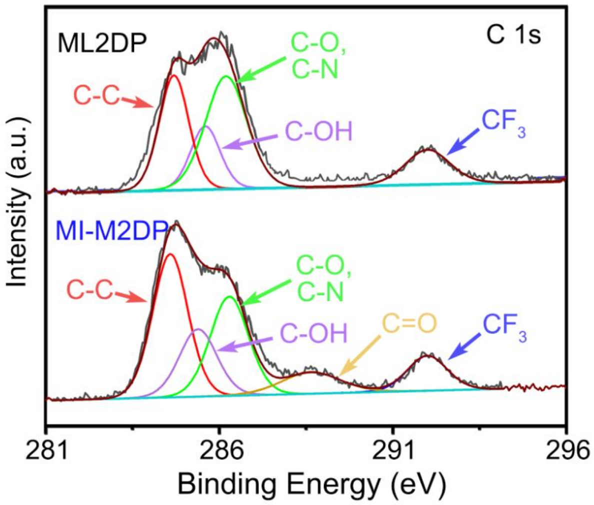

Supplementary Fig. 20. Curve-fitted high-resolution XPS C 1s spectra of **ML2DP** and **MI-M2DP**.

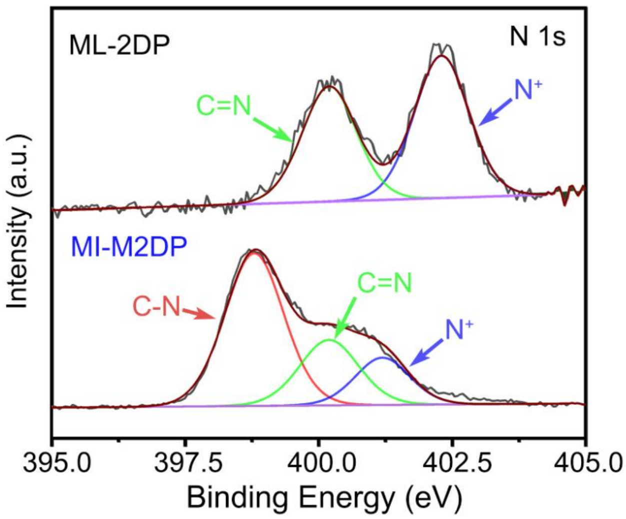

Supplementary Fig. 21. Curve-fitted high-resolution XPS N 1s spectra of **ML2DP** and **MI-M2DP**.

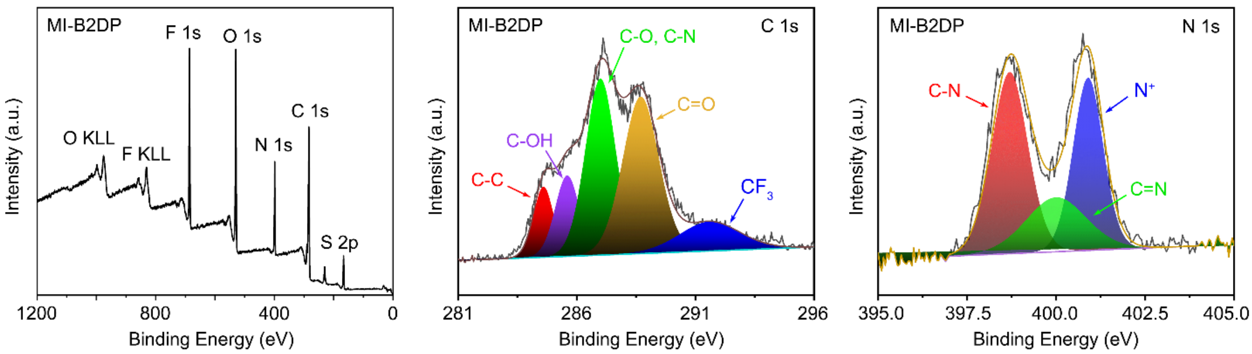

Supplementary Fig. 22. XPS survey scan spectrum, curve-fitted high-resolution XPS C 1s and N 1s spectra of **MI-B2DP**.

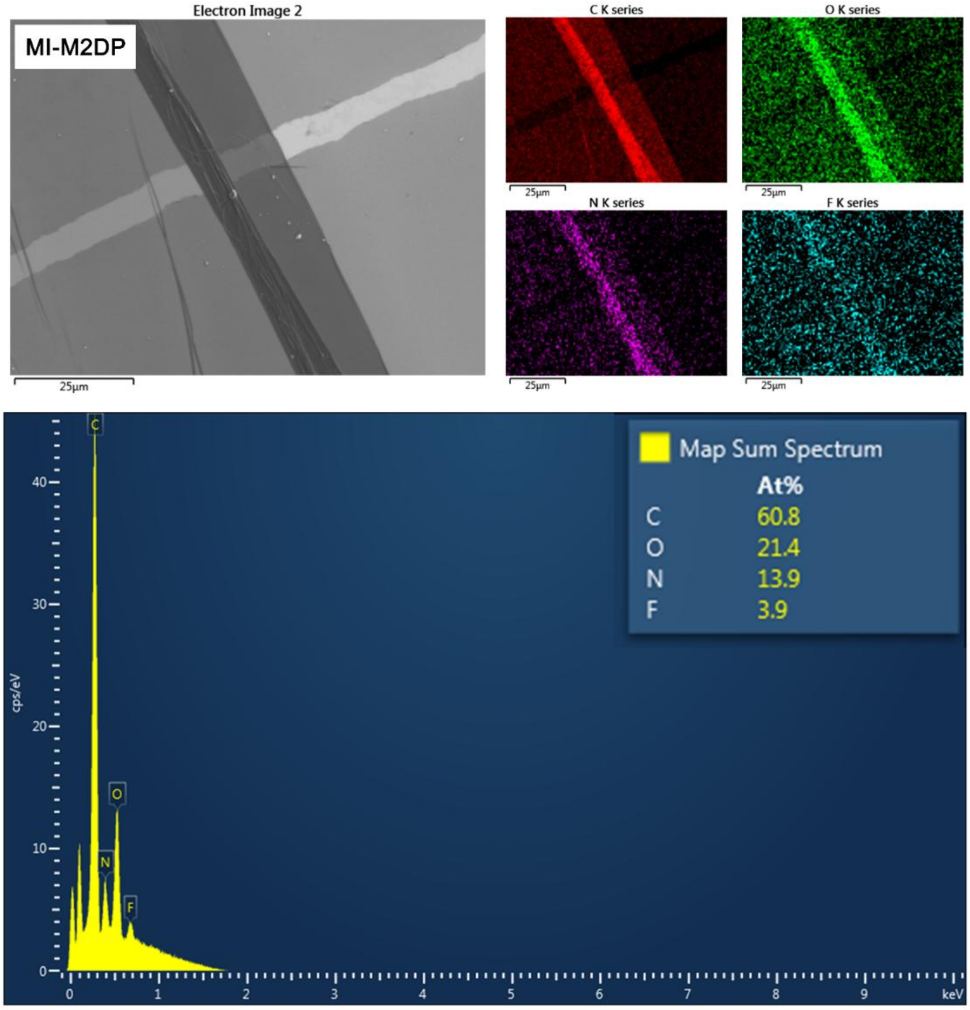

Supplementary Fig. 23. SEM image and EDX mapping pattern of **MI-M2DP**.

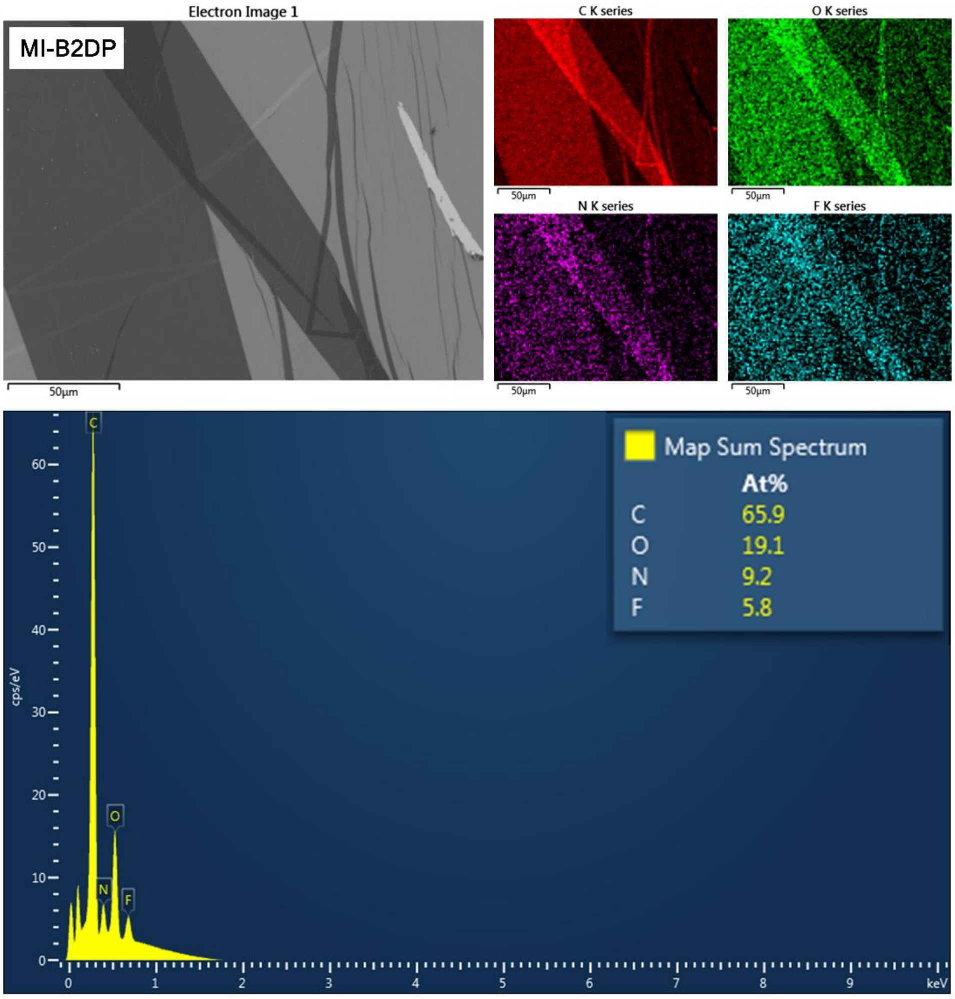

Supplementary Fig. 24. SEM image and EDX mapping pattern of **MI-B2DP**.

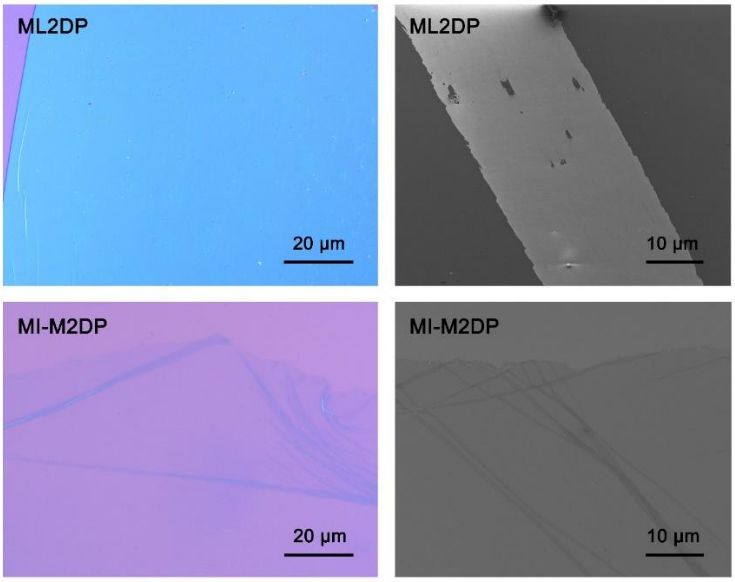

Supplementary Fig. 25. OM and SEM images of **ML2DP** and **MI-M2DP** on SiO_2/Si substrates.

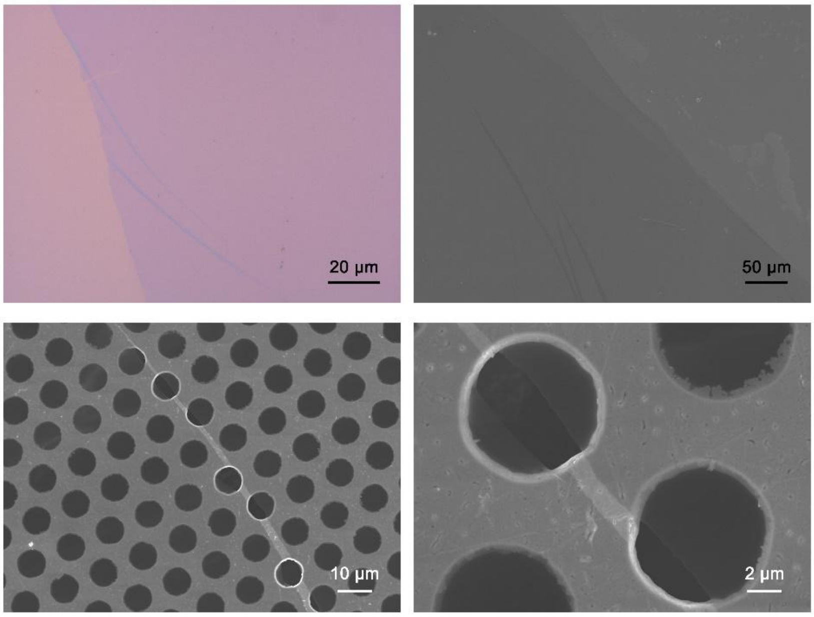

Supplementary Fig. 26. OM and SEM images of **MI-B2DP** on SiO_2/Si substrates. SEM and high-resolution SEM images of **MI-B2DP** on copper grids with a hole diameter of ~5 μm.

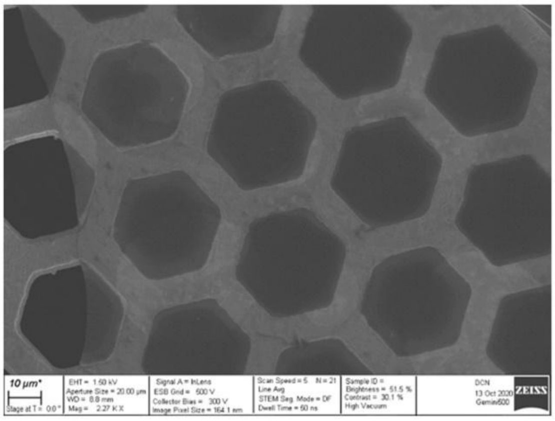

Supplementary Fig. 27. SEM image of **ML2DP** on a copper grid with a hole diameter of ~20 µm.

To assess whether the incorporation of **MCMs** affects the surface roughness of the resulting 2DP films, we analyzed the surface morphology of **ML2DP** (without **MCMs**), **MI-M2DP** (with **MCMs**) and **MI-B2DP** (with **MCMs**) films (Supplementary Fig. 28). The root mean square roughness (Rq) values for **ML2DP**, **MI-M2DP**, and **MI-B2DP** were determined to be 0.21 nm, 0.18 nm, and 0.27 nm, respectively, while their corresponding arithmetic average roughness (Ra) values were 0.16 nm, 0.11 nm, and 0.22 nm. These results indicate no significant difference in surface roughness between the **MCM**-containing and **MCM**-free 2DP films. However, the absolute accuracy of the roughness analysis is limited by the finite radius of the AFM tip, which can underestimate local variations, especially in nanoporous films.

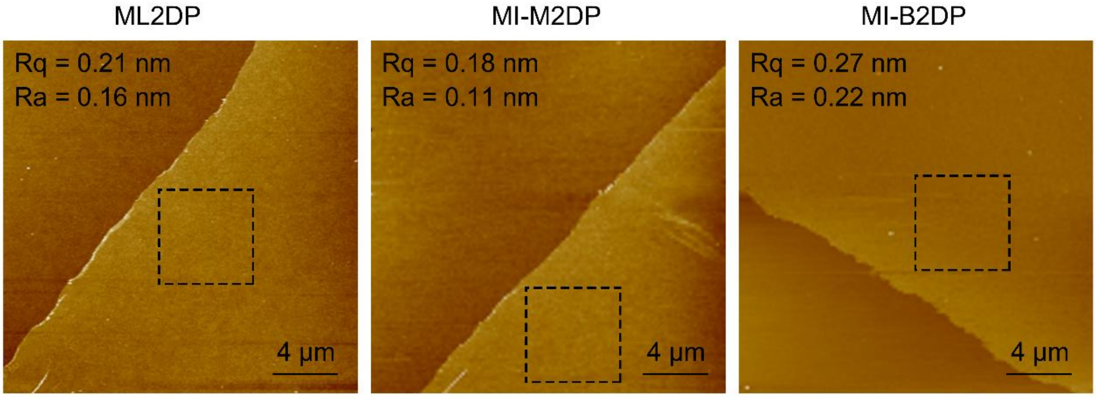

Supplementary Fig. 28. AFM images and the corresponding Rq and Ra values of **ML2DP**, **MI-M2DP** and **MI-B2DP**.

The thickness was measured by AFM in tapping mode on films transferred onto SiO_2/Si substrates. While these 2DPs contain periodic nano-holes (~4.5 nm), the AFM tip radius (~8 nm) is insufficient to resolve such nanopores within the network. Therefore, the measured step height between the 2DP-covered and uncovered regions provides an estimate of the average vertical dimension of the continuous molecular framework.

To further support the observed thickness difference between **MI-M2DP** and **MI-B2DP** films, we have now included their AFM height profiles, histogram and bearing area plots, as shown in Supplementary Figs. 29 and 30. The histograms of **MI-M2DP** and **MI-B2DP** films reveal distinct height distributions centered at ~1.7 and ~2.1 nm, respectively. Notably, only 1.7% area of the **MI-M2DP** reaches 2.1 nm, confirming the meaningful difference in thickness between **MI-M2DP** and **MI-B2DP** films.

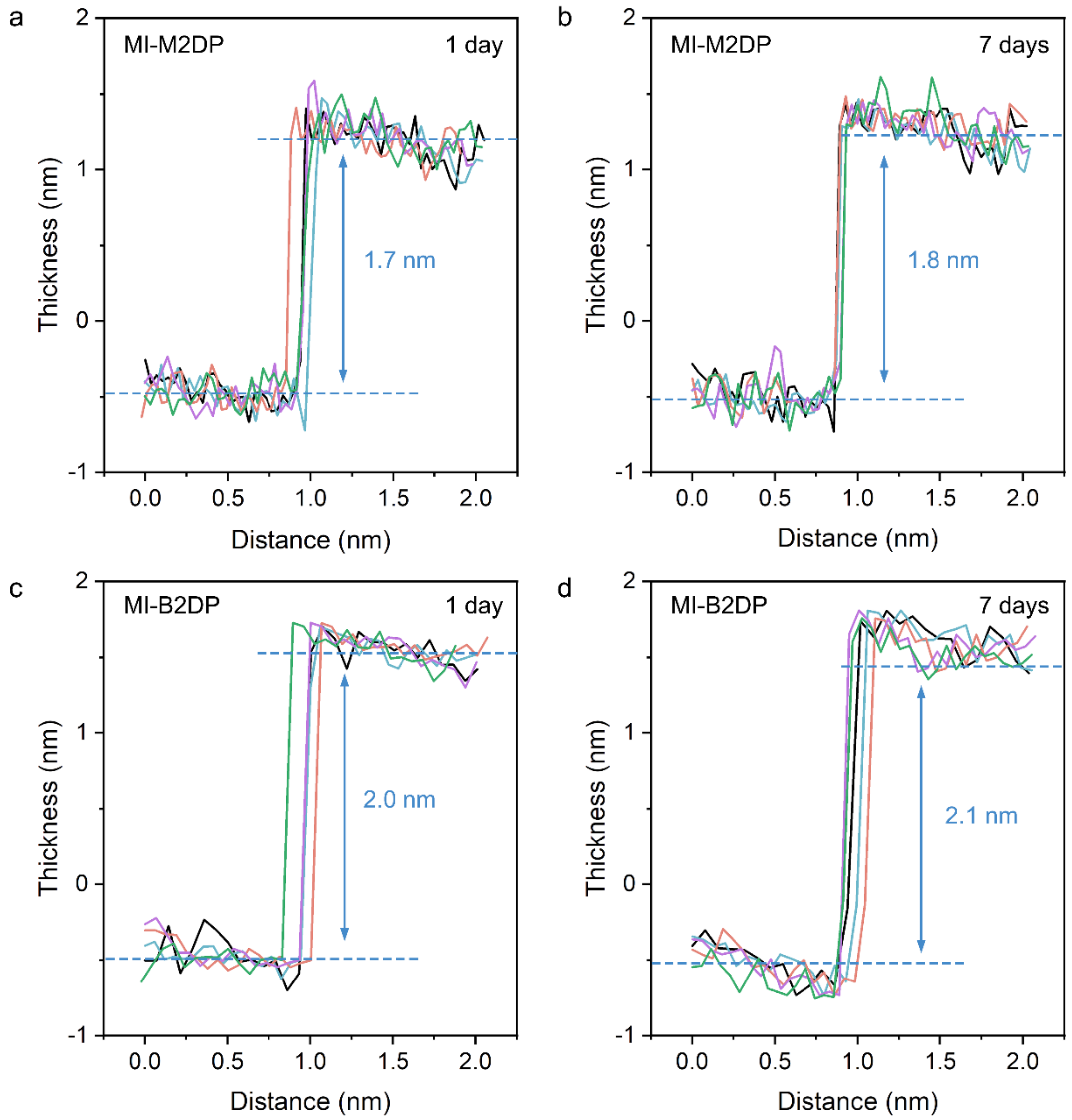

Supplementary Fig. 29. a,**b**, AFM height profiles of **MI-M2DP** film after 1 day (**a**) and 7 days (**b**) of polymerization. **c**,**d**, AFM height profiles of **MI-B2DP** film after 1 day (**c**) and 7 days (**d**) of polymerization.

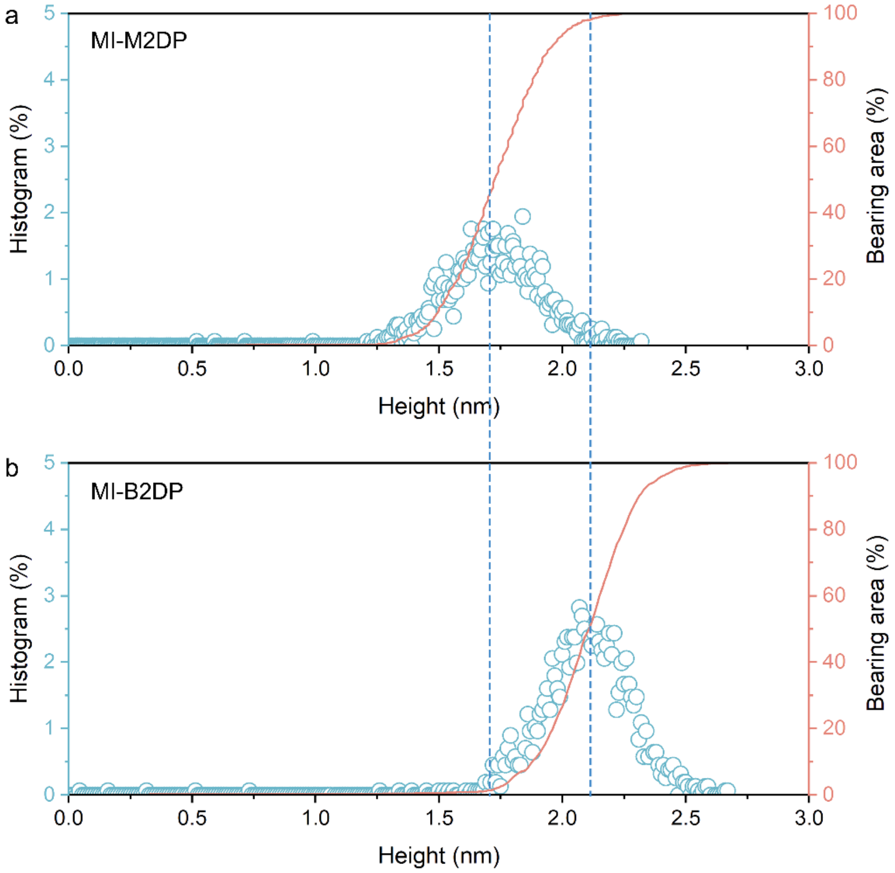

Supplementary Fig. 30. a,**b**, Height histograms and bearing area curves of **MI-M2DP** (**a**) and **MI-B2DP** (**b**) films.

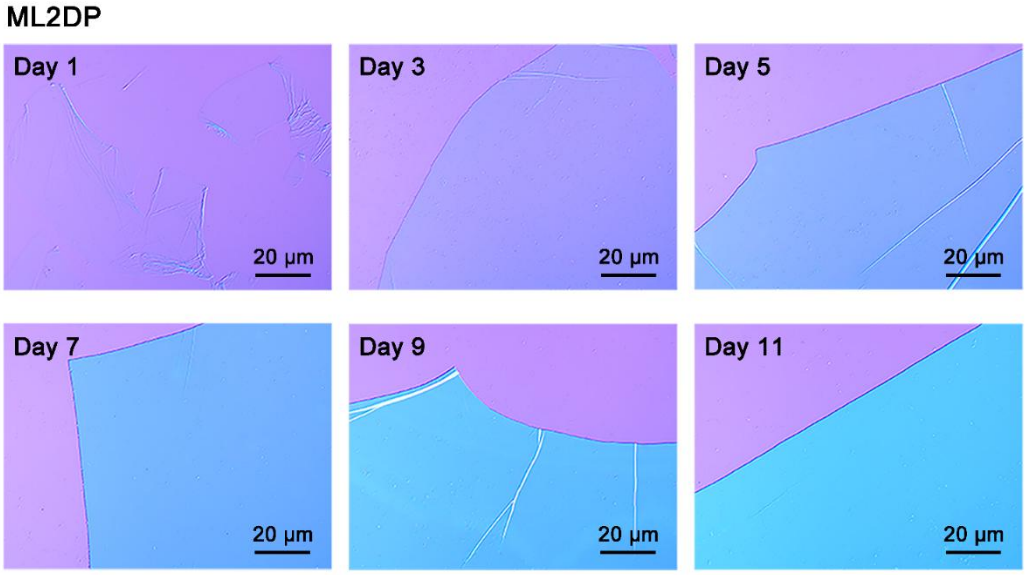

Supplementary Fig. 31. OM images of **ML2DP** on SiO_2/Si substrates with respect to reaction time.

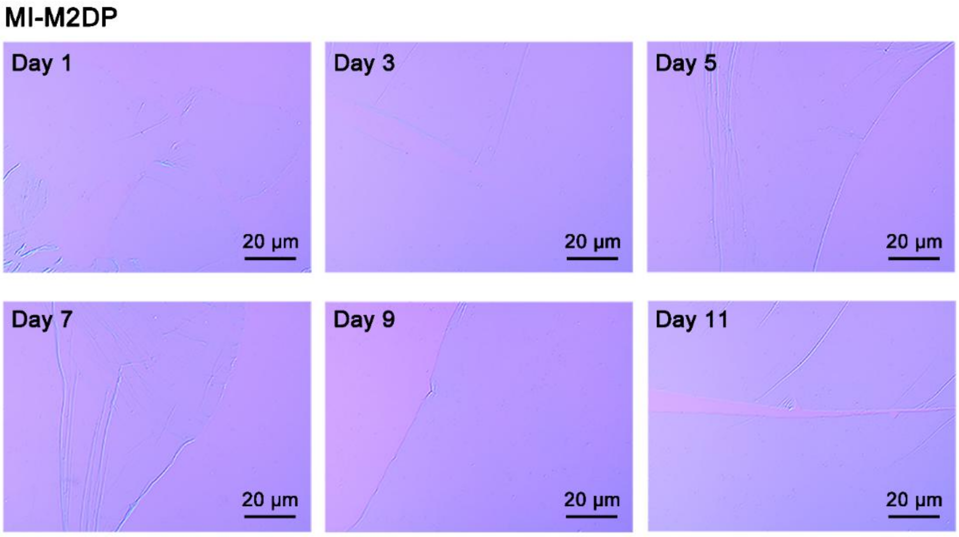

Supplementary Fig. 32. OM images of **MI-M2DP** on SiO_2/Si substrates with respect to reaction time.

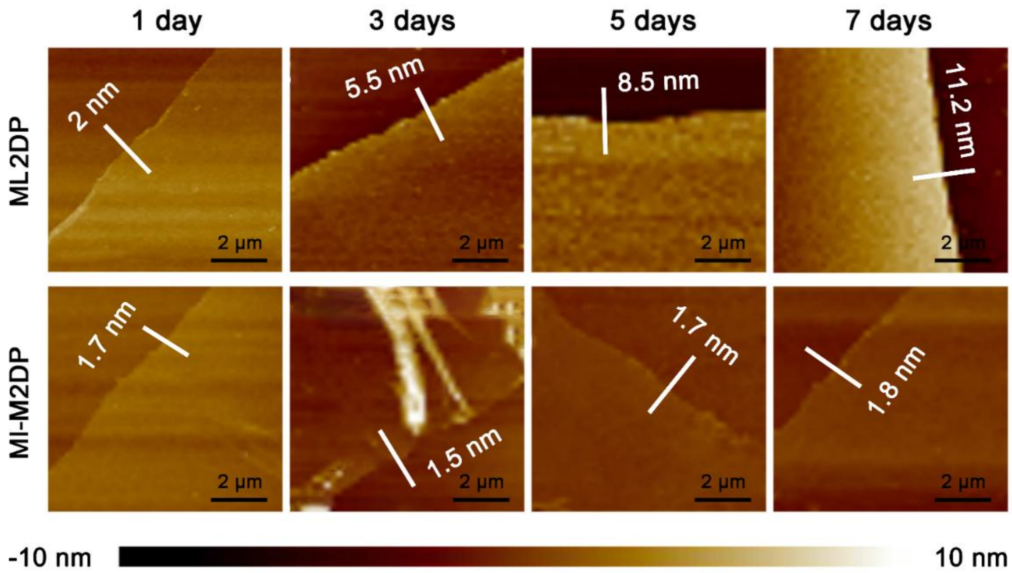

Supplementary Fig. 33. AFM images of **ML2DP** and **MI-M2DP** on a SiO_2/Si substrate with respect to reaction time.

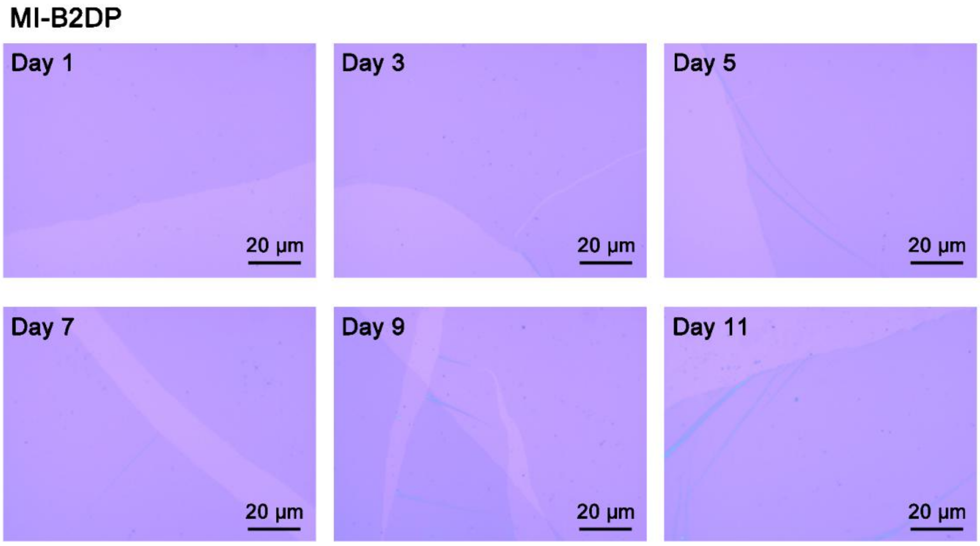

Supplementary Fig. 34. OM images of **MI-B2DP** on SiO_2/Si substrates with respect to reaction time.

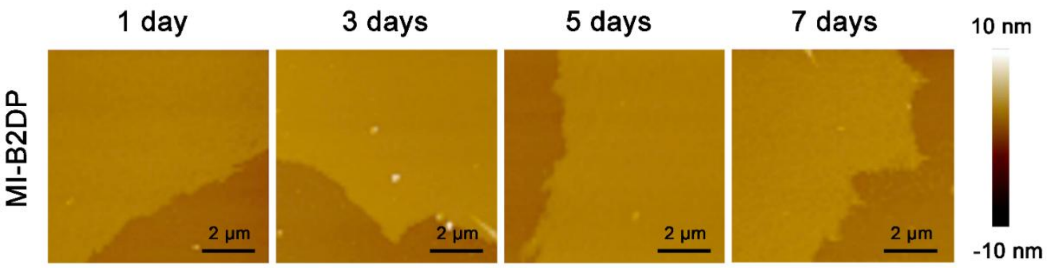

Supplementary Fig. 35. AFM images of **MI-B2DP** on SiO_2/Si substrates with respect to reaction time.

To further substantiate the role of the SOS monolayer, we have conducted a control experiment in its absence (Supplementary Fig. 36). After 1 day of polymerization, no discernible 2D polymer film was observed, in stark contrast to the experiments conducted with the SOS monolayer. This result highlights the critical role of the SOS monolayer in guiding monomer absorption, preorganization, and promoting 2D polymerization on the water surface.

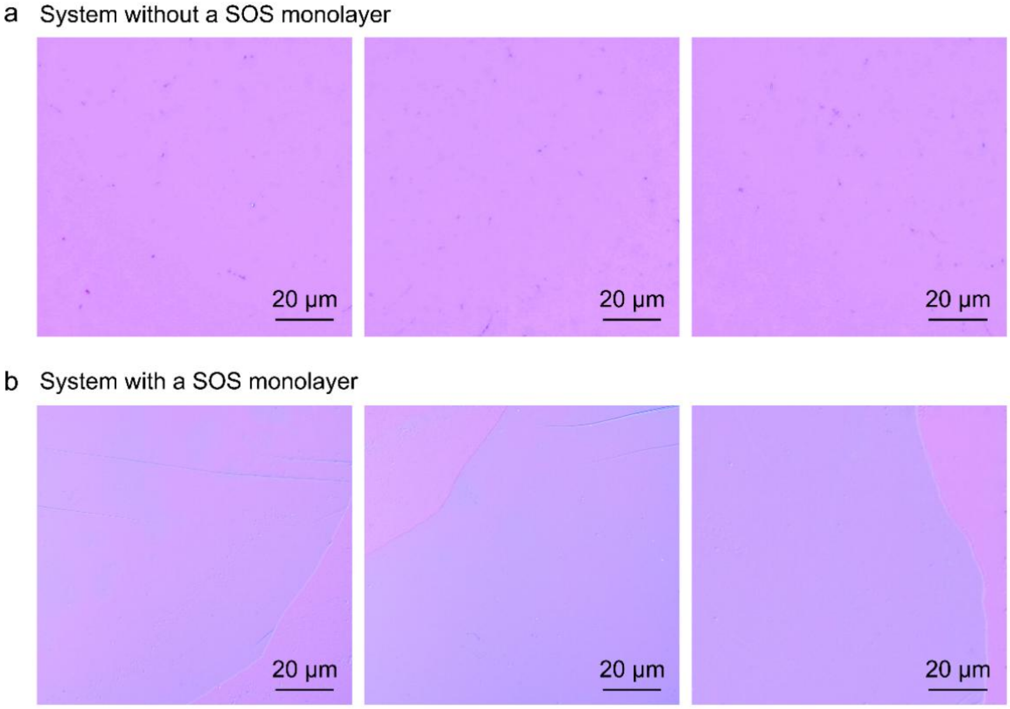

Supplementary Fig. 36. a, OM images of products transferred from the system without a SOS monolayer. **b**, OM images of products transferred from the system with a SOS monolayer.

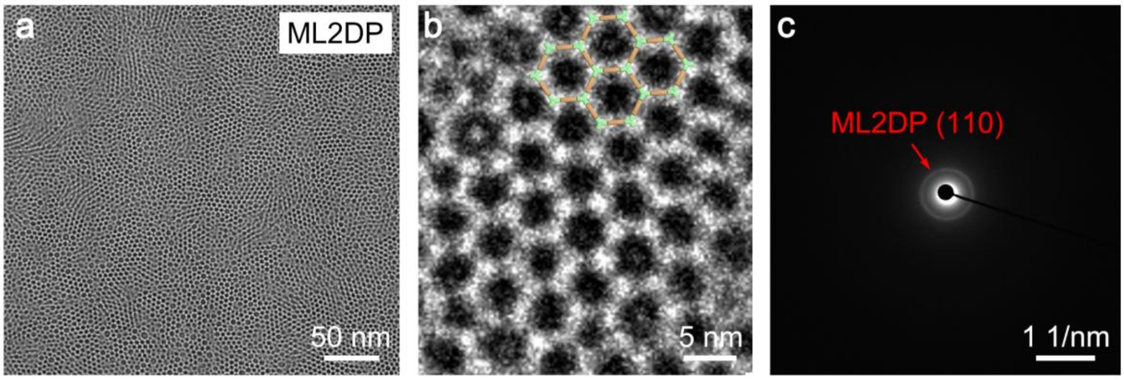

Supplementary Fig. 37. High-resolution TEM images and SAED pattern of **ML2DP**. High-resolution TEM images and corresponding fast Fourier transform (FFT) pattern reveal that **ML2DP** film exhibits a crystalline structure with periodic hexagonal pores with an in-plane lattice of $a = b = 44.5$ Å.

MI-M2DP or **MI-B2DP** was encapsulated between two monolayer graphene to form a protective sandwich structure (Supplementary Fig. 38). Monolayer graphene on Cu foil (Graphenea Deutschland, GmbH) was first floated on a 0.1 M ammonium persulfate (APS) solution for 8 h to etch away the underlying Cu foil, with the PMMA side facing upward. The resulting PMMA/graphene film was transferred to Milli-Q water surface to thoroughly remove residual etchant, then placed onto a TEM grid and dried. The PMMA was removed by immersing the grid in acetone for 4 hours, followed by rinsing with isopropanol and drying, yielding a clean bottom graphene layer. The free-standing 2DP was then transferred onto the graphene-coated grid, rinsed with isopropanol. After drying, the second graphene monolayer was transferred on top using the same procedure, completing the protective sandwich structure.

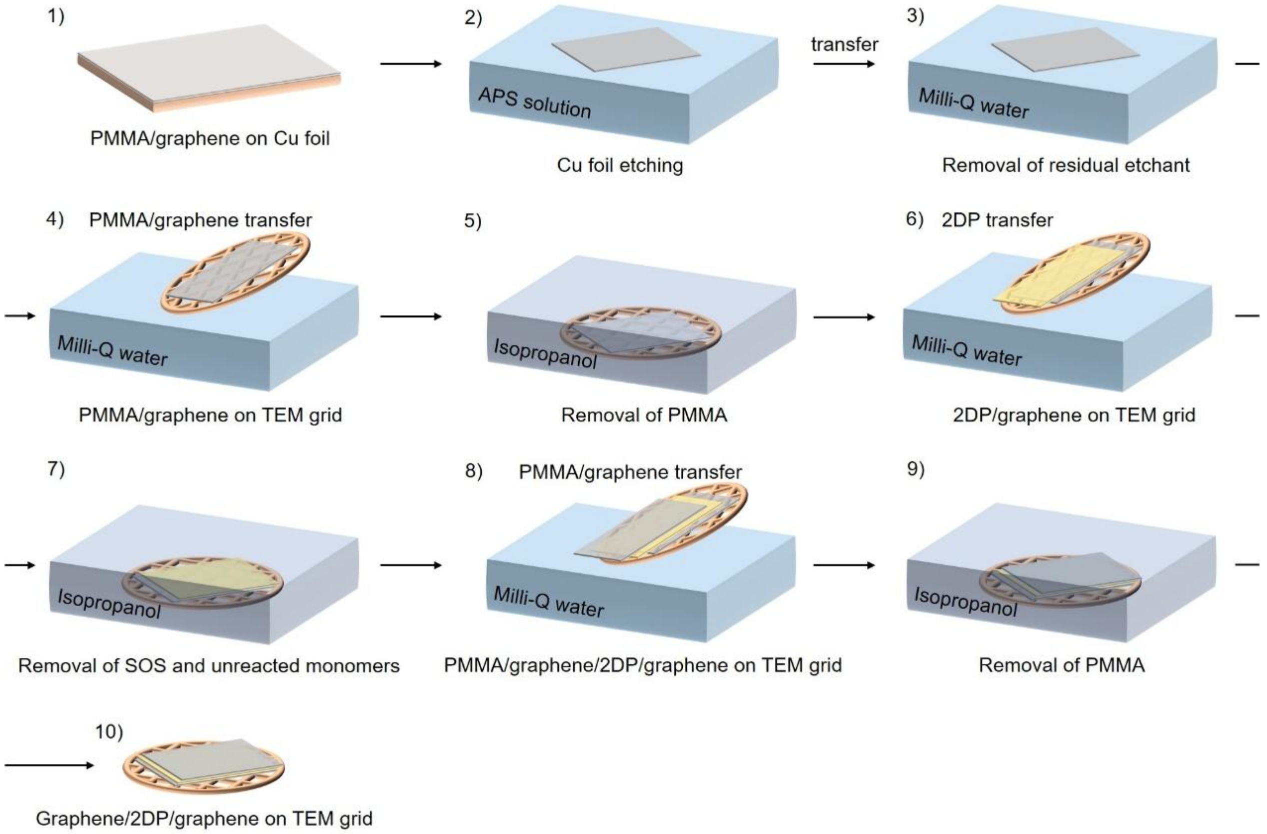

Supplementary Fig. 38. Schematic for the fabrication of the graphene/2DP/graphene sandwich structure.

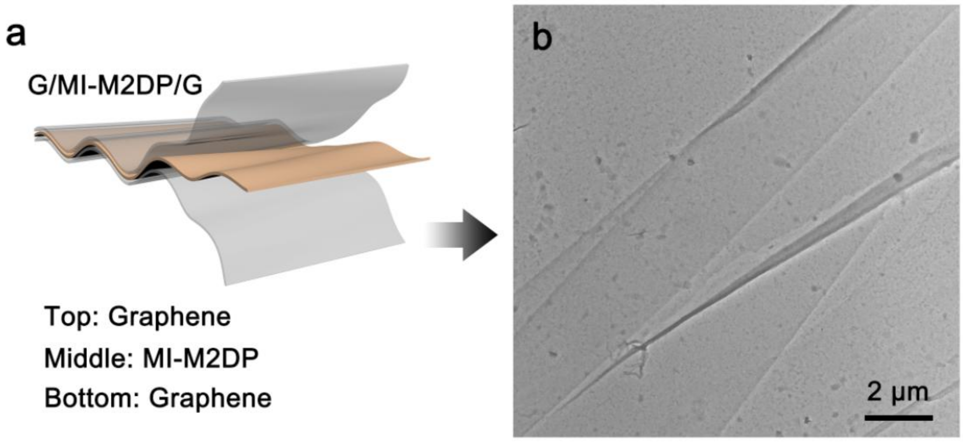

Supplementary Fig. 39. a, Schematic for the formation of the sandwich structure **G/MI-M2DP/G**. **b,** TEM image of the **G/MI-M2DP/G** sandwich structure.

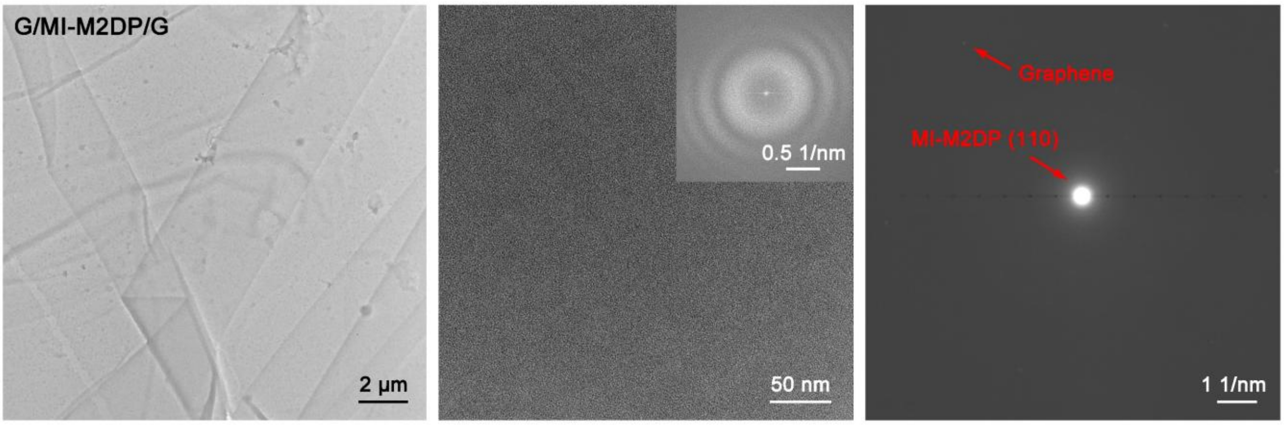

Supplementary Fig. 40. TEM images and SAED pattern of the **G/MI-M2DP/G**. Diffractions at 0.45 nm^{-1} (i.e. *d*-spacing of 22.2 Å) and 4.35 nm^{-1} (i.e. *d*-spacing of 2.3 Å) correspond to the (110) plane of **MI-M2DP** and the first order reflection of graphene, respectively.

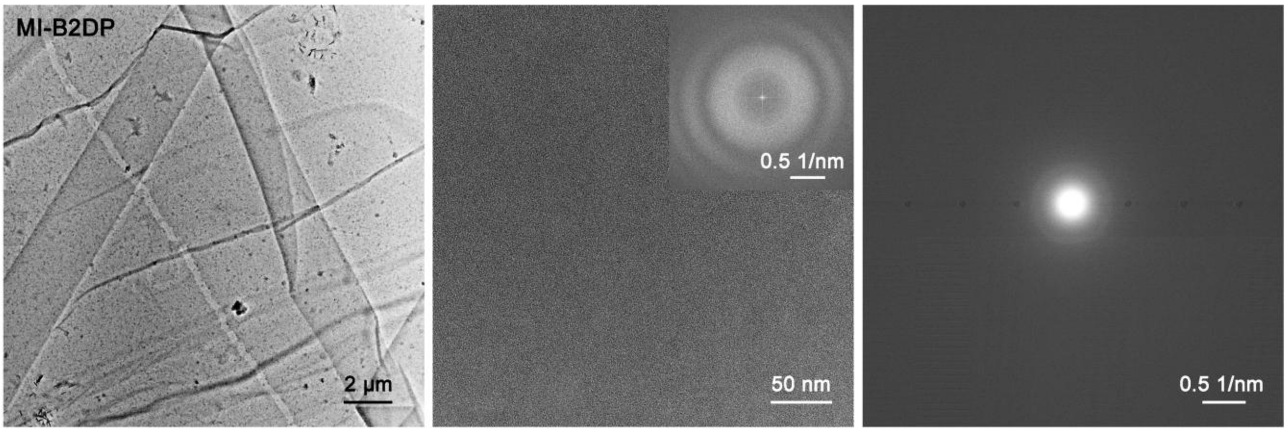

Supplementary Fig. 41. TEM images and SAED pattern of **MI-B2DP**. Diffraction ring at 0.45 nm^{-1} (i.e. *d*-spacing of 22.2 Å) corresponds to the (110) plane of **MI-B2DP**.

The multilayer 2DP films have stacked layers as protective cushions, which are more resilient and tolerant to electron beam and X-ray irradiation, thus facilitating high-resolution structural characterization at the molecular or atomic level. In contrast, the ultra-thin feature of monolayer and bilayer 2D polymers leaves the entire structure fully exposed to the high energy beam. The radiation-induced knock-on damage, electrostatic charging, and chemical etching result in their lattice distortions or complete degradation,[3,4] posing significant challenges for X-ray and TEM analysis.[5] Thereby, the GIWAXS and TEM diffraction patterns can be observed exclusively upon the stacking of additional graphene or 2D polymer layers as shown in Supplementary Fig. 42.

To capture the high-quality HRTEM images, we lowered the voltage to 80 kV and applied a low electron dose rate of ~0.2 e^{-} $Å^{-2}$ s^{-1} for the TEM measurements. Under these conditions, the hexagonal structure of **MI-M2DP** and **MI-B2DP**, with a lattice parameter of a = b = 44.5 Å, γ = 120°, was successfully resolved in the HRTEM images (Fig. 2b,f), agreeing well with the SAED and GIWAXS results. Multiple HRTEM images taken at different positions further confirmed the polycrystalline nature of both **MI-M2DP** and **MI-B2DP** (Supplementary Fig. 44). However, due to inevitable beam damage, achieving molecular resolution that allows for distinguishing framework components remains challenging.

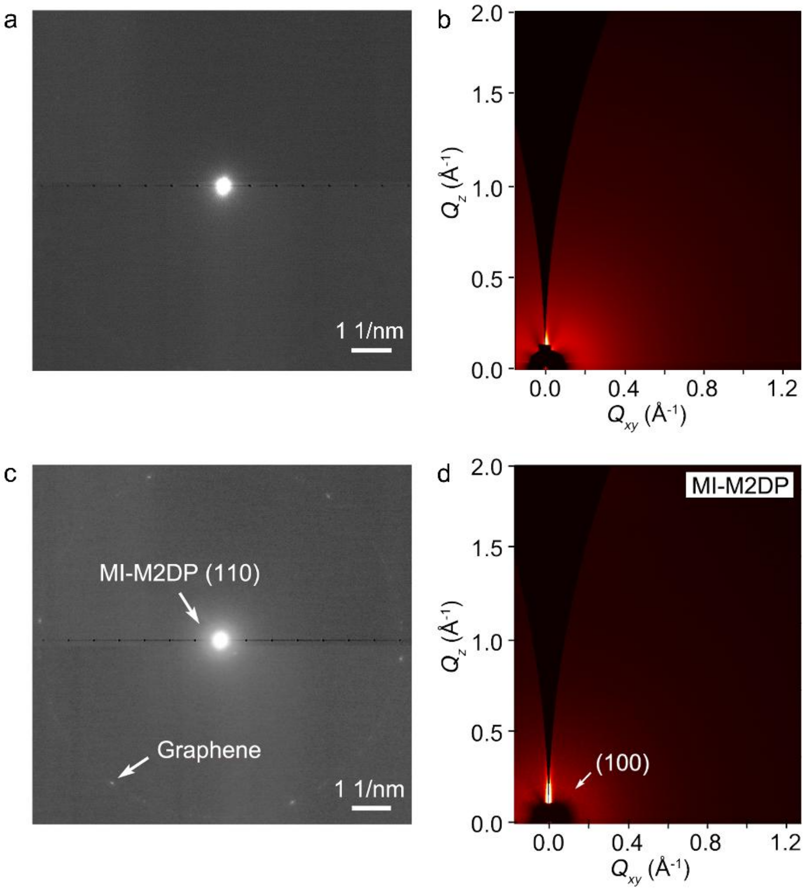

Supplementary Fig. 42. a, SAED pattern of MI-M2DP. **b**, GIWAXS pattern of MI-M2DP. **c**, SAED pattern of G/MI-M2DP/G. **d**, GIWAXS pattern of a 20-layer MI-M2DP film. Note that MI-M2DP suffers from lattice distortions or complete degradation under the electron beam and X-ray irradiation, leading to undetectable SAED and GIWAXS signals.

As shown in Supplementary Fig. 43, the GIWAXS azimuthal intensity profile of the (100) reflections for **MI-M2DP** and **MI-B2DP** reveal broad distributions (predominantly within the 0-60° range), indicative of the random in-plane orientation. However, we would like to highlight that this distribution cannot reflect the intrinsic crystallographic orientation of **MI-M2DP** and **MI-B2DP**. Note that to avoid beam damage, GIWAXS measurements were performed on ~20-layer stacks assembled via layer-by-layer transfer. During the transfer process, structural imperfections such as wrinkles, undulations, and misalignments can be introduced, which compromise the overall orientational coherence and obscure the intrinsic alignment of individual layers in the azimuthal analysis.

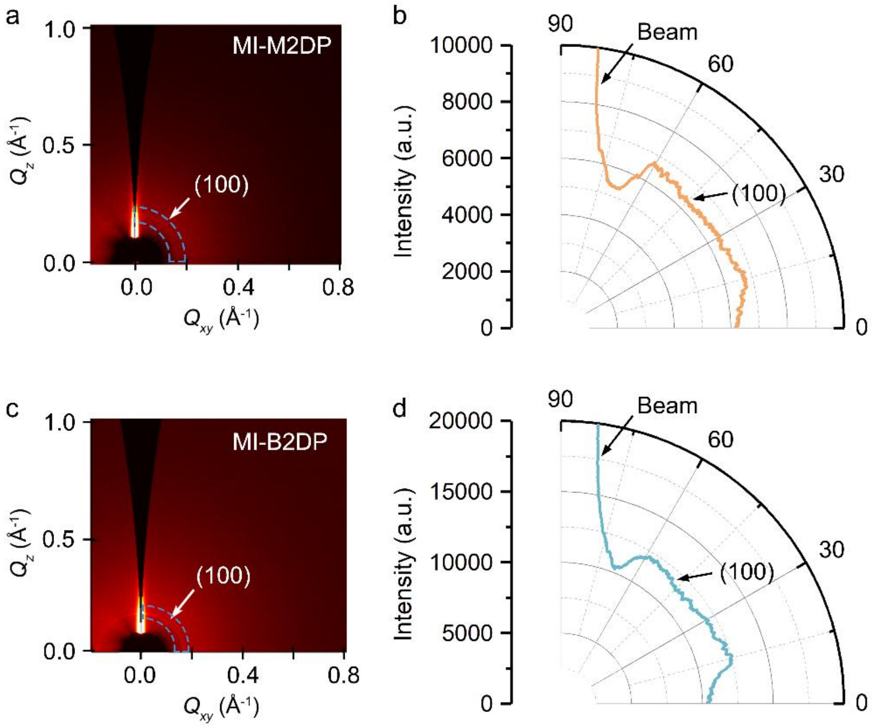

Supplementary Fig. 43. **a**,**b**, GIWAXS pattern (**a**) and azimuthal intensity distribution of the (100) diffraction peak (**b**) for the **MI-M2DP** film. **c**,**d**, GIWAXS pattern (**c**) and azimuthal intensity distribution of the (100) diffraction peak (**d**) for the **MI-B2DP** film. Q represents the integrated scattering vector of in-plane (Q_{xy}) and out-of-plane (Q_z).

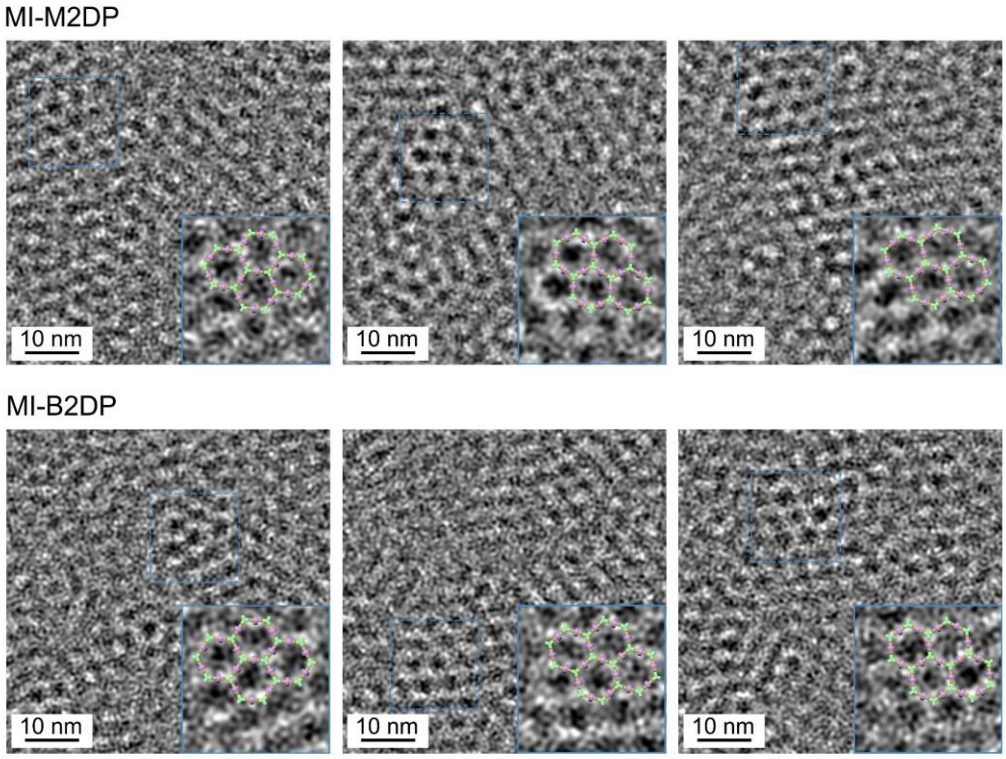

Supplementary Fig. 44. HRTEM images of **MI-M2DP** and **MI-B2DP**. Insets: Magnified HRTEM images with the honeycomb structure overlaid. The polycrystalline **MI-M2DP** and **MI-B2DP** films consist of multiple crystalline domains and some amorphous regions.

The SIEBIMM method offers a rapid, non-destructive approach to estimate the in-plane Young's modulus and strain-dependent behavior of ultrathin films. It relies on the formation of periodic buckling patterns when a film adhered to a pre-strained elastomeric substrate (e.g., PDMS) is released. By analyzing the wrinkle wavelength and amplitude, the mechanical modulus can be derived. However, this method is sensitive to uncertainties in the film thickness and substrate modulus, and it provides limited abilities to assess fracture behavior and strength. In contrast, AFM nanoindentation is a widely adopted and quantitatively robust technique for characterizing the local mechanical properties, particularly effective for suspended films, where substrate effects can be minimized. By indenting the film with a calibrated tip and recording the load-displacement response, both Young's modulus and breaking strength can be extracted. Nevertheless, this method requires careful calibration of AFM tip geometry and force sensitivity; therefore, it is more time-consuming and may introduce artifacts if the film exhibits inhomogeneity or if the tip radius is comparable to structural features of the material.

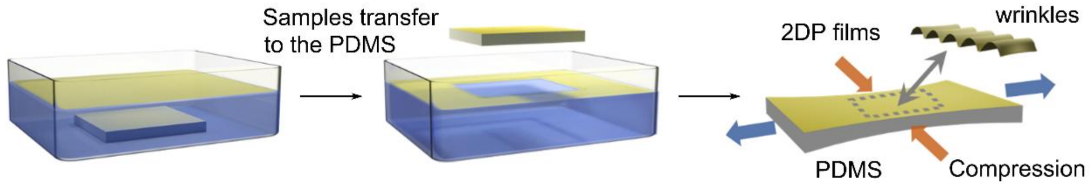

Supplementary Fig. 45. Schematic illustration of the transfer of 2DP films onto a plain PDMS and the lateral compression of PDMS during uniaxial transverse stretching, inducing monolayer wrinkling.

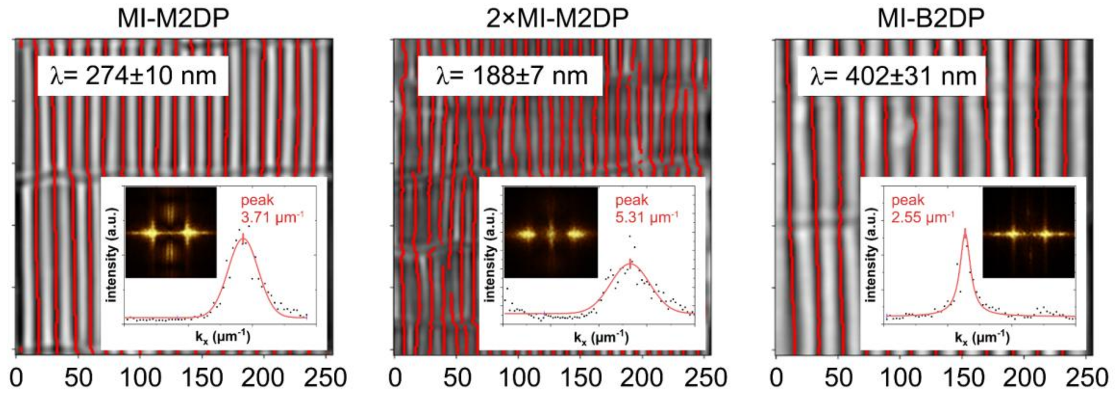

Supplementary Fig. 46. Wavelength calculation images for **MI-M2DP**, **2×MI-M2DP,** and **MI-B2DP** based on the line-wise wavelength and amplitude calculation process. Integrated intensity profile after 2D Fourier-transformation along k_x direction (insert).

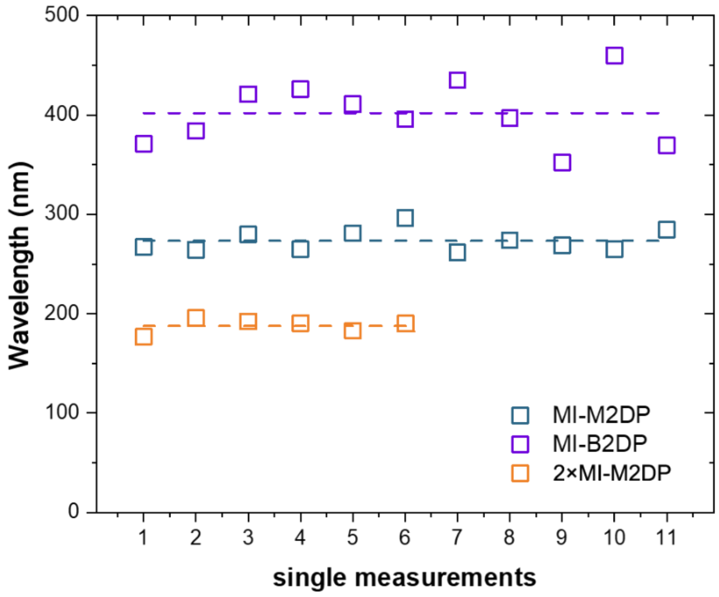

Supplementary Fig. 47. Wrinkles wavelengths of **MI-M2DP**, **2×MI-M2DP** and **MI-B2DP** with different single measurements.

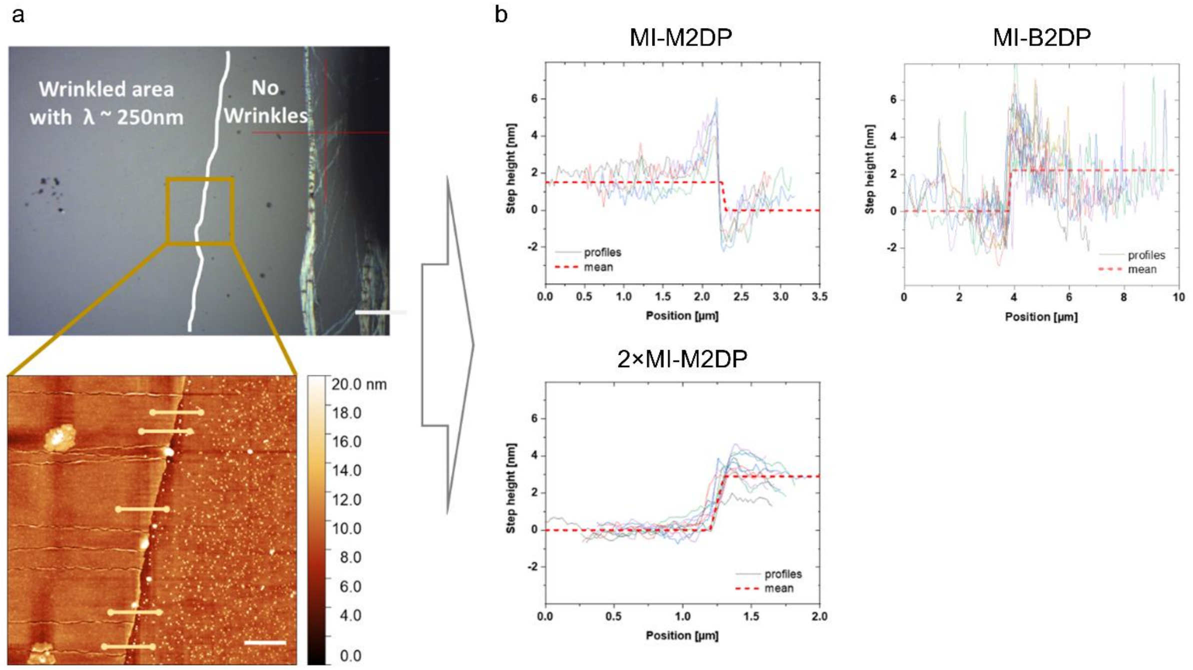

Supplementary Fig. 48. Example procedure of thickness control. **a**, Search for adjacent wrinkled and non-wrinkled areas under 10% compression and step height measurement at these positions after strain release to erase the wrinkles. **b**, Extraction of step height profiles and analysis with step height fitting using the open source SPM analyze software Gwyddion (Ver. 2.61).

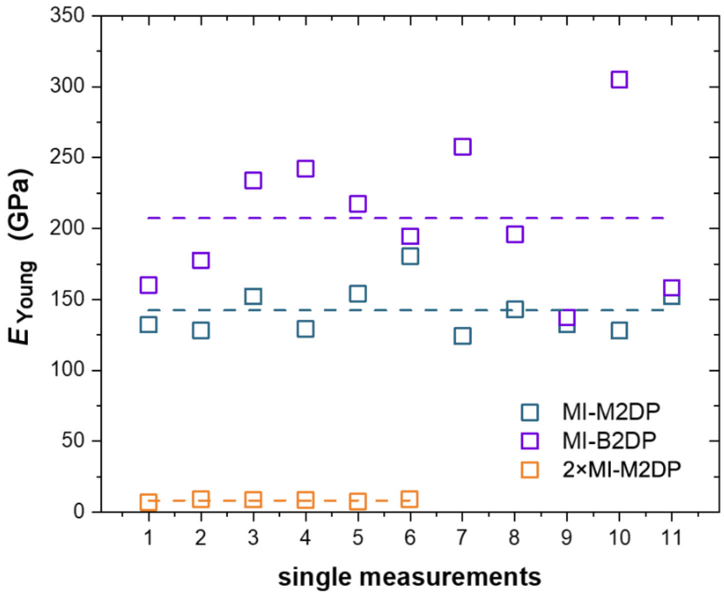

Supplementary Fig. 49. E_{Young} of **MI-M2DP**, **2×MI-M2DP** and **MI-B2DP** with different single measurements.

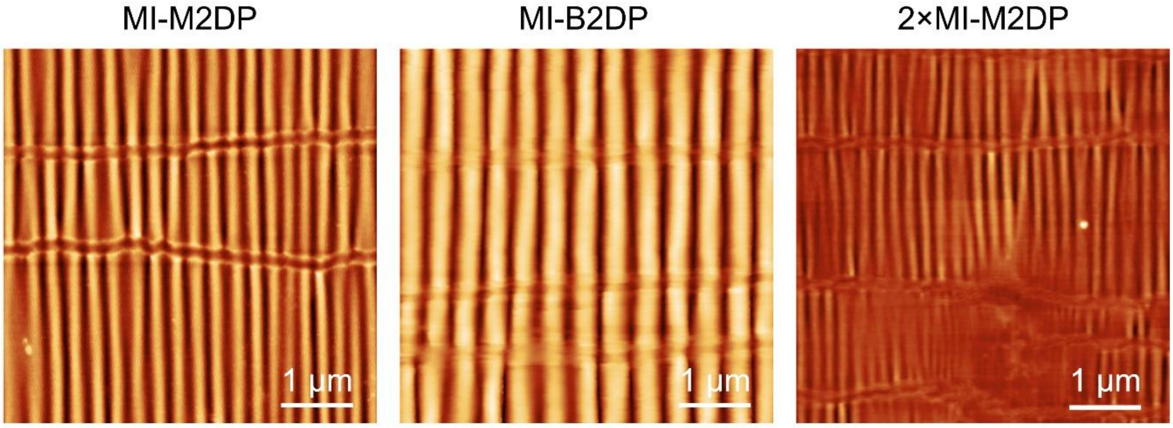

Supplementary Fig. 50. High-resolution AFM images of **MI-M2DP**, **MI-B2DP** and **2×MI-M2DP**.

To evaluate the interlayer behavior during mechanical property measurement, we further performed images morphology analysis. The high-resolution AFM images display well-ordered wrinkle patterns in **MI-M2DP** and **MI-B2DP**, indicating strong interlayer adhesion. In contrast, a locally irregular pattern was observed in **2×MI-M2DP**, revealing interlayer sliding and decoupling during compression (Supplementary Fig. 50). Furthermore, the obtained wavelength versus thickness dependency $\lambda(h)$ also enables to act as a qualitative indicator to characterize the interlayer adhesion behavior of multilayer samples. The wavelengths of **MI-M2DP** and **MI-B2DP** follow a linear and orthogonal $\lambda(h)$ relation (blue line, Supplementary Fig. 51a) as the converted wrinkling equation.[6]

$$\lambda = 2\pi h \left(\frac{E_{\text{Young\_}f}/(1-\nu_f^2)}{3\,E_{\text{Young\_}s}/(1-\nu_s^2)} \right)^{1/3}$$

This phenomenon provides evidence for the robust interlayer coupling in **MI-B2DP**, which effectively constrains both the in-plane and out-of-plane motion of the bilayer, thereby influencing the wrinkling process. In contrast, the vdW-stacked **2×MI-M2DP**, with a thickness of ~3.0 nm, exhibits an opposing behavior characterized by a significant reduction in wavelength due to weak interlayer adhesion. During compression, the bottom layer of the **2×MI-M2DP**, which is well adhered to the PDMS substrate, initiates wrinkling, resulting in a wavelength comparable to that of the **MI-M2DP** film. However, the vdW-stacked top layer of **2×MI-M2DP** does not contribute to the wrinkling process. Instead, it undergoes mechanical relaxation due to interlayer sliding, merely following the given wrinkled surface without delamination in the best case. The same behavior was observed for the vdW-stacked multilayer **MI-M2DP** (~5-30 layers) films as their thicknesses increase from ~3.0 to 18.1 nm, leading to the pronounced differences in calculated E_{Young} (Supplementary Fig. 51b). These results demonstrate the significant impact of interlayer interactions on the mechanical properties of 2D materials.

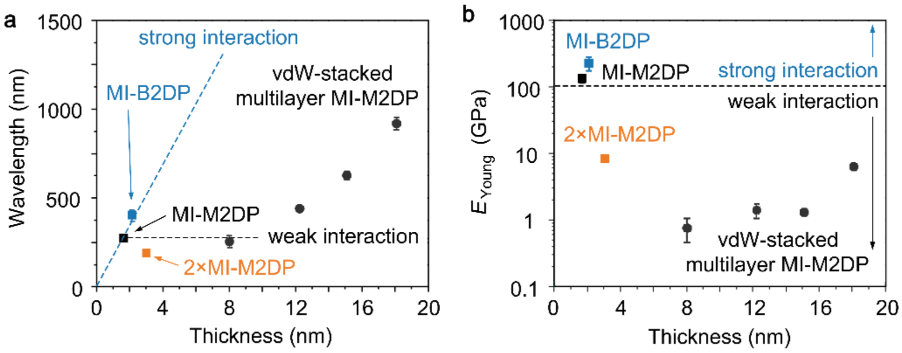

Supplementary Fig. 51. Thickness-dependent wavelength results λ(h) (**a**) and E_{Young} (**b**) for **MI-M2DP**, **MI-B2DP**, vdW-stacked **2×MI-M2DP**, and vdW-stacked multilayer **MI-M2DP** (~5-30 layers) at 10% compressive strain. All values are expressed as mean ± SD, n=6.

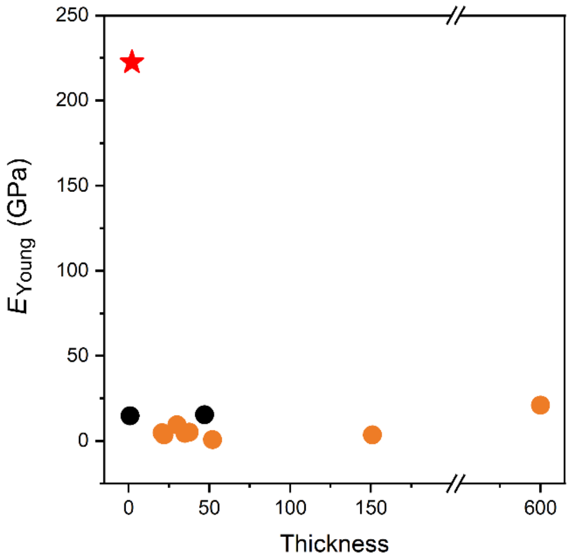

Supplementary Fig. 52. Mechanical property comparison of **MI-B2DP** with thus-far reported layer-stacked organic framework films and carbon nanomembranes (CNM) measured by the SIEBIMM method.

Traditional AFM nanoindentation typically refers to localized surface deformation in bulk or supported materials. However, this technique has been widely adapted to characterize suspended thin films, where the stress response is governed by out-of-plane deflection under a localized point load[7-9]. In these studies, a free-standing film is suspended over microfabricated apertures or trenches, and the AFM tip applies a calibrated normal force at the center of the suspended region. The resulting force-displacement curve reflects the film's bending or stretching response, enabling the extraction of key mechanical parameters such as Young's modulus, pre-tension, and fracture strength. Despite the differences in deformation mechanics, the term "AFM nanoindentation" remains commonly used due to its operational similarity and the use of indentation-style force mapping.

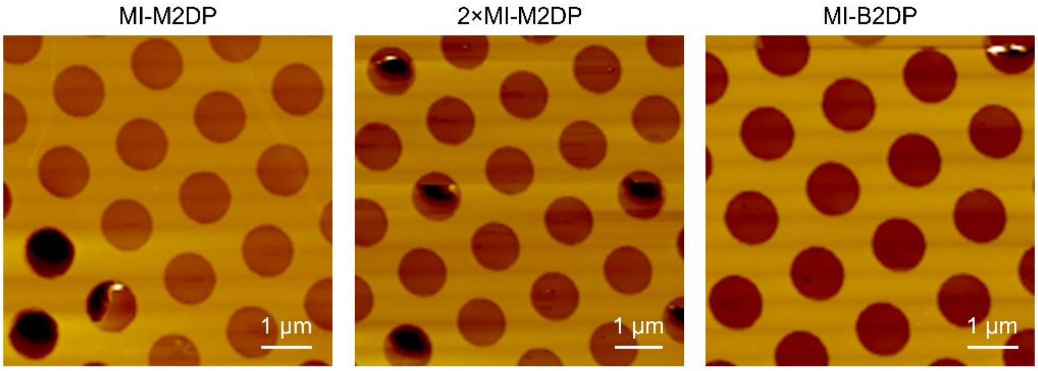

Supplementary Fig. 53. AFM images of the **MI-M2DP**, **2×MI-M2DP** and **MI-B2DP** films suspended over circular holes with a diameter of 1 μm.

The **2×MI-M2DP** films were prepared through a layer-by-layer transfer of **MI-M2DP** on the substrates twice. The resulting **2×MI-M2DP** samples are characterized by random stacking. Yet, considering the negligible interlayer interactions of **2×MI-M2DP** (weak van der Waals interaction), the misaligned stacking does not significantly affect the mechanical property. To explore the impact of stacking modes on mechanical properties, we calculated the in-plane elasticity of AA and AB-stacked **2×MI-M2DP**, considering the largest difference in offsets (0 vs. 25.4 Å). The same slope was observed in the stress-strain curves, revealing that their E_{Young} remains consistent (Supplementary Fig. 54).

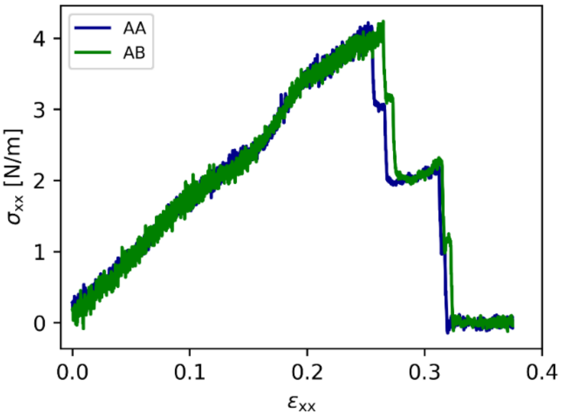

Supplementary Fig. 54. Calculated in-plane stress-strain curves along zig-zag (xx) direction of the **2×MI-M2DP** films with AA (blue) and AB (green) stacking.

We prepared 3 samples of **2×MI-M2DP** for the SIEBIMM characterizations and 5 samples for the AFM nanoindentation characterizations, respectively, where each sample was measured more than twice at different positions. We would expect all these samples to have different stacking modes. As shown in Supplementary Fig. 55, the E_{Young} values calculated by these parallel experiments were distributed around 8±5 and 46±11 GPa, within the normal error range. These results demonstrate that the impact of the stacking modes on mechanical properties is negligible.

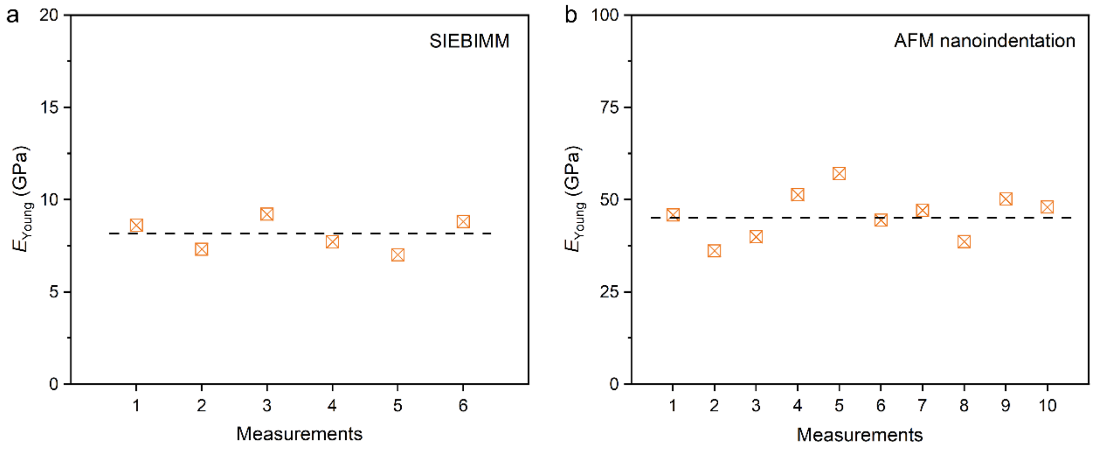

Supplementary Fig. 55. E_{Young} values of the **2×MI-M2DP** film measured by the SIEBIMM (**a**) and AFM nanoindentation (**b**) methods.

We employed a standard AFM nanoindentation method to investigate the fracture behavior. The load vs. deflection (F-δ) curves and AFM images of **MI-M2DP**, **2×MI-M2DP** and **MI-B2DP** films were recorded up to fracture as shown in Fig. 4d. After the indentation, the films remained suspended on the hole, with nanoscale fracture occurring only in the area direct contact with the AFM tip (Supplementary Fig. 61). The films did not exhibit any additional cracks that are typically induced by the internal defects under stress loading (Supplementary Fig. 56), indicating the consistent fracture behavior across the different 2DP films.

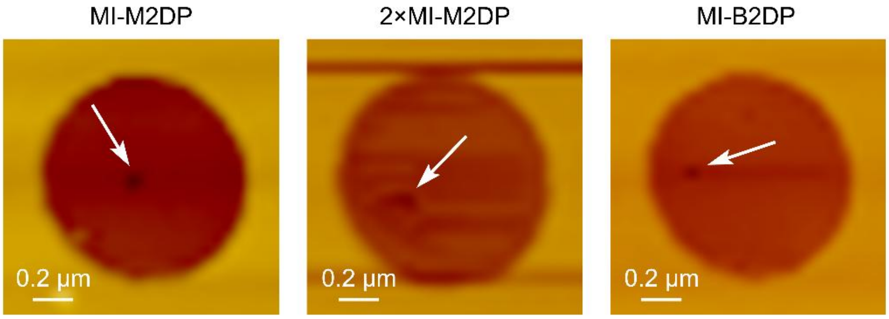

Supplementary Fig. 56. AFM images of **MI-M2DP**, **2×MI-M2DP** and **MI-B2DP** films after the indentation.

The 2DP films are representative by the polycrystalline nature, which contain the microscopic defects. To further investigate the effect of these defects on the fracture behavior, we simulated the unit cell of **MI-M2DP** with linker defects in the armchair and zig-zag directions, as shown in Supplementary Fig. 57. The stress-strain curves of **MI-M2DP** without any defect (Supplementary Fig. 58) and with defects in the armchair (Supplementary Fig. 59) and zig-zag (Supplementary Fig. 60) directions were calculated. Note that, despite a 50% defect concentration in the **MI-M2DP** structure, the fracture points (ε_{xx}: 0.34 and ε_{yy}: 0.31 for the armchair defect structure; ε_{xx}: 0.33 and ε_{yy}: 0.37 for the zig-zag defect structure) are comparable with the pristine system (ε_{xx}: 0.32 and ε_{yy}: 0.26). These results demonstrate that the internal defects in **MI-M2DP** do not have significant effects on the fracture process.

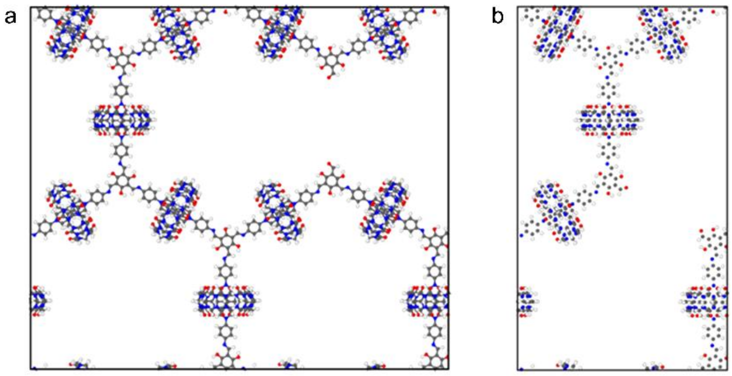

Supplementary Fig. 57. Simulated unit cell of **MI-M2DP** with the linker defects in the armchair (**a**) and zig-zag (**b**) directions.

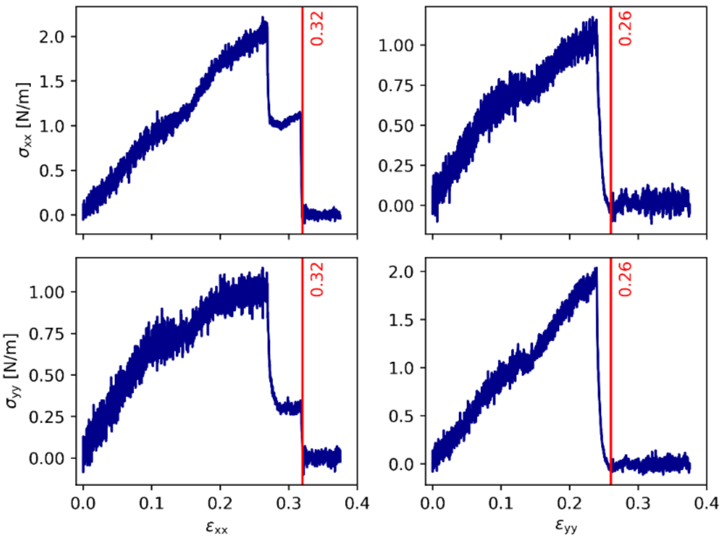

Supplementary Fig. 58. Stress strain curves along the zig-zag (xx) and armchair (yy) directions for **MI-M2DP** without any defect. The red vertical lines indicate the strain at which the system is fully ruptured.

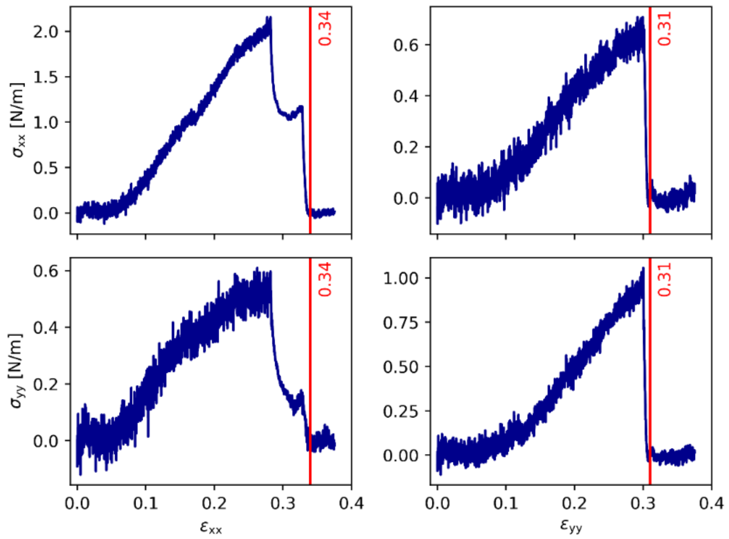

Supplementary Fig. 59. Stress strain curves along the zig-zag (xx) and armchair (yy) directions for **MI-M2DP** with linker defects in the armchair direction. The red vertical lines indicate the strain at which the system is fully ruptured.

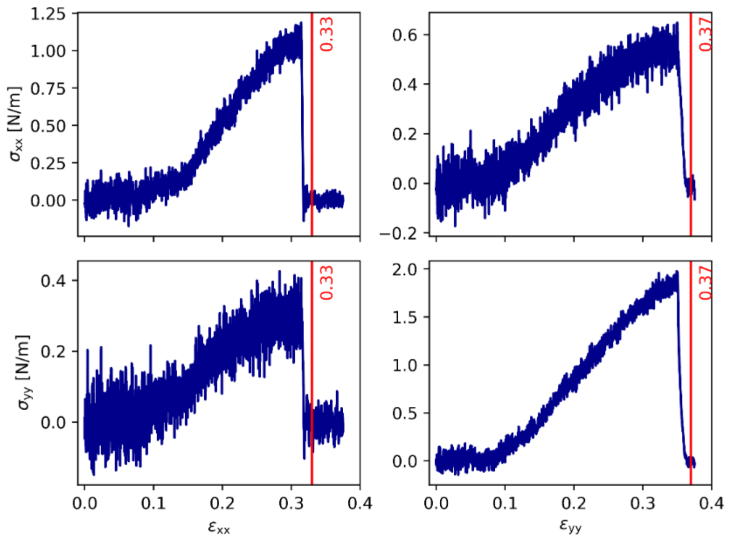

Supplementary Fig. 60. Stress strain curves along the zig-zag (xx) and armchair (yy) directions for **MI-M2DP** with linker defects in the zig-zag direction. The red vertical lines indicate the strain at which the system is fully ruptured.

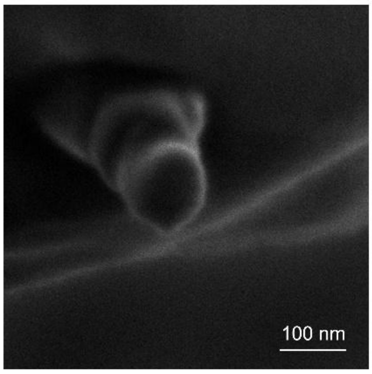

Supplementary Fig. 61. The SEM image of the AFM tip with a radius of ~13.5 nm.

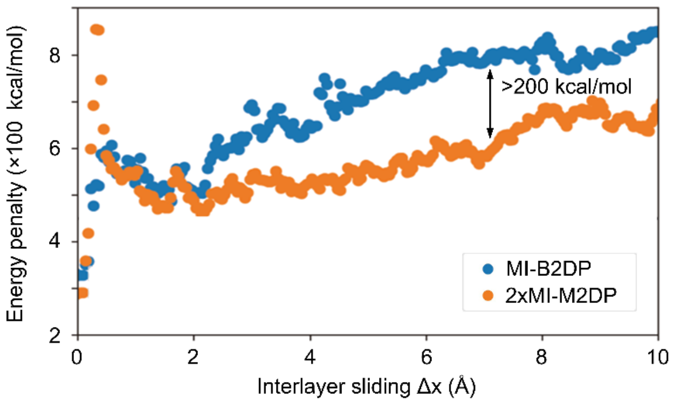

Supplementary Fig. 62. Energy barrier of **MI-B2DP** and **2×MI-M2DP** upon the interlayer sliding.

We calculated the in-plane 2D elastic modulus (E^{2D}) for **MI-M2DP**, **2×MI-M2DP** and **MI-B2DP** as shown in the stress-strain curves (Figs. 5c and Supplementary Figs. 63-67). The top layer of **2×MI-M2DP** was weakly coupled to the bottom layer, enabling its free movement during the stress loading. This results in a negligible contribution to the stress response from the top layer. To simulate this, we constructed a discontinuous layer on another **MI-M2DP** layer, making the top layer free to move during the stretching. But for **MI-B2DP**, we modeled the mechanically interlocked two layers, which are tightly combined and stretched equally. The theoretical E^{2D} values of **MI-M2DP**, **2×MI-M2DP** and **MI-B2DP** were (219±4) 10^{-2}, (232±2) 10^{-2} and (454±3) 10^{-2} N m^{-1}, respectively. Thereby, the Young's modulus (E) of **MI-M2DP**, **2×MI-M2DP** and **MI-B2DP** were calculated by the equation $E = E^{2D}/h$. Compared to **MI-M2DP** ((129±2) 10^{-2} GPa), the E of **MI-B2DP** ((216±1) 10^{-2} GPa) increased by 67%, while the E of **2×MI-M2DP** ((77±1) 10^{-2} GPa) decreased by 40%. It is important to note that the structures used for modeling only reflect an idealized model of the single crystalline framework with high porosity, which present different atomic structure to the as-prepared films. This difference between the calculation and experiment leads to the deviation of the obtained E values.

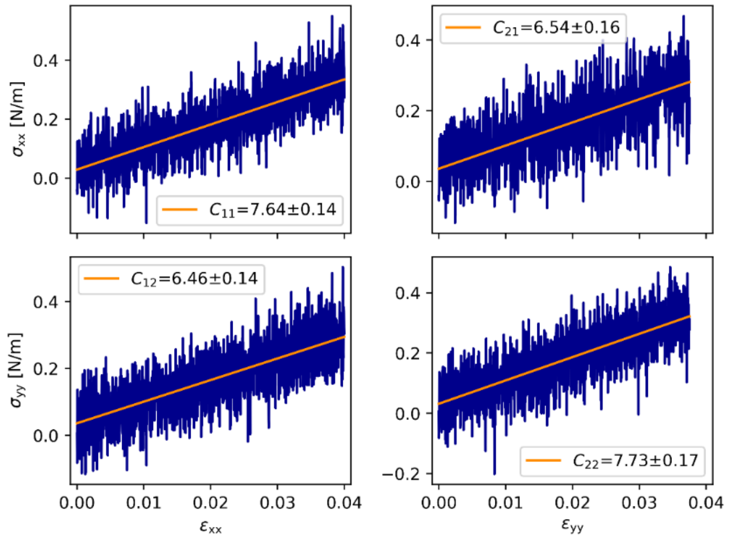

Supplementary Fig. 63. Simulated stress-strain curves of **MI-M2DP** (E^{2D} = (219±4) 10^{-2} N m^{-1}).

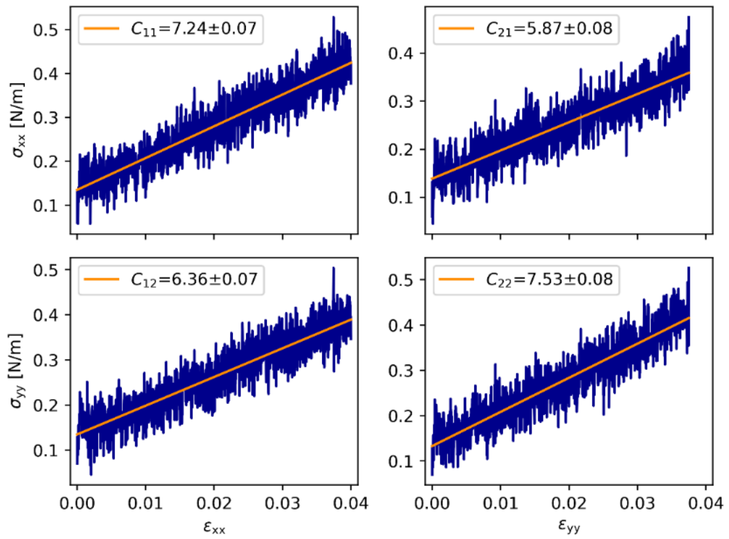

Supplementary Fig. 64. Simulated stress-strain curves of **2×MI-M2DP**.

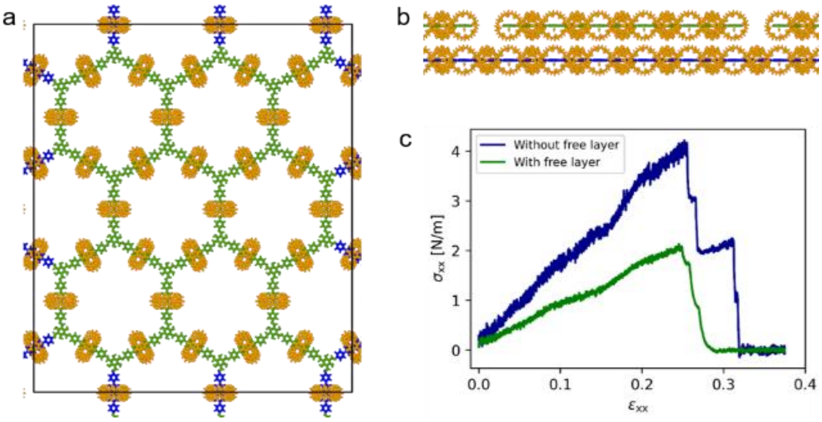

Supplementary Fig. 65. a,**b**, Simulation setup of **2×MI-M2DP**. **c**, Comparison of the stress strain curve along the zig-zag (xx) direction for **2×MI-M2DP** with (green, E^{2D} = (232±2) 10^{-2} N m^{-1}) and without (blue, E^{2D} = (578±2) 10^{-2} N m^{-1}) a free moving top layer. By comparing these two scenarios, the sliding of the top layer was observed and its contribution to the overall stress response is negligible

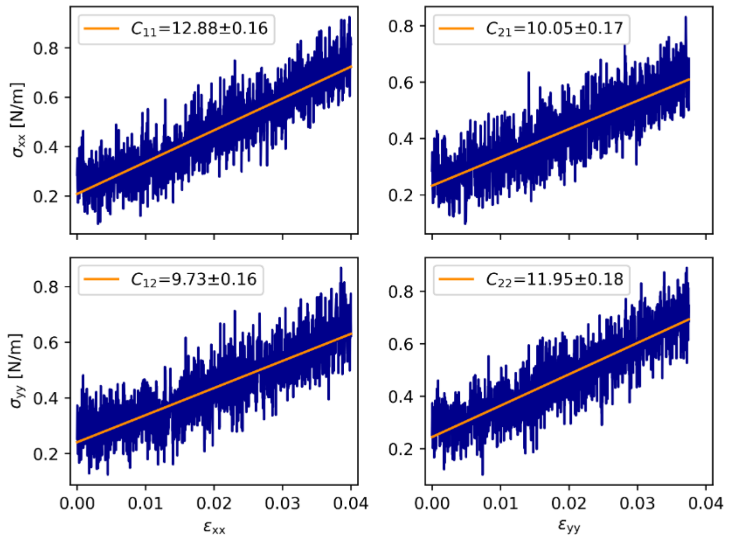

Supplementary Fig. 66. Simulated stress-strain curves of **MI-B2DP**.

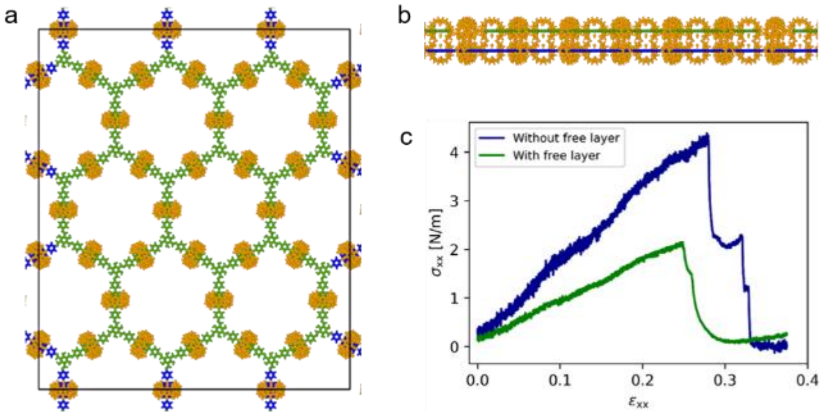

Supplementary Fig. 67. a,**b**, Simulation setup of **MI-B2DP**. **c**, Comparison of the stress strain curve along the zig-zag (xx) direction for MI-B2DP with (green, E^{2D} = (264±2) 10^{-2} N m^{-1}) and without (blue, E^{2D} = (454±3) 10^{-2} N m^{-1}) a free moving top layer. However, in the nanoindentation experiment, the two layers of **MI-B2DP** are strongly coupled by the interlocked structure. Thereby, there is no free moving top layer in this interlocked system (blue curve).

Supplementary Table 1. The element content of C, N, O, S, F in **ML2DP** and **MI-M2DP** calculated by XPS data.

	C (at. %)	N (at. %)	O (at. %)	S (at. %)	F (at. %)
**ML2DP**	62.9	5.7	19.5	3.1	8.8
**MI-M2DP**	53.7	18.5	17.6	3.7	6.5

Supplementary Table 2. Summarized averaged wavelength and thickness values based on different measurement spots and the averaged calculation result for the in-plane E_{Young} of each 2DP.

Sample	λ [nm]	h [nm]	E_{Young} [GPa]
**MI-M2DP**	274 ± 10	1.7 ± 0.1	130 ± 27
**MI-B2DP**	402 ± 31	2.1 ± 0.1	222 ± 60
**2×MI-M2DP**	188 ± 7	3.0 ± 0.6	8 ± 5

Supplementary Table 3. Comparison of the modulus and breaking strength of 2D COFs, 2D MOFs and CNMs.

Materials		Methods	Modulus (GPa)	Breaking strength (GPa)	Reference
2D Polymers & 2D COFs	MI-B2DP	Nanoindentation	151.4	19	**This work**
	MI-B2DP	Buckling	222.4	N/A	
	TAPB-PDA	Nanoindentation	42.5	3.66	9
			40.5	3.3	
	TAPB-DMTP	Nanoindentation	45.6	4.59	
			48.8	4.83	
	TTA-DHTA	Nanoindentation	26	N/A	10
	Tp-Azo	Nanoindentation	15.3	N/A	11
	Tp-DPP	Nanoindentation	6.35	N/A	
	TPB-BOP	Nanoindentation	2.72	0.0385	12
	TPPy-BOP	Nanoindentation	N/A	0.0256	
	TPB-F-BOP	Nanoindentation	4.4	N/A	
	2DPA-1	Nanoindentation	12.7	0.488	7
	FCOF	Nanoindentation	30	N/A	13
2D MOFs	CuBDC	Nanoindentation	23	N/A	14
	$[Cu(\mu\text{-}pym_2S_2)(\mu\text{-}Cl)]_n$	Nanoindentation	5	1	15
	Cu-BHT	Nanoindentation	0.98	N/A	16
	PdTCPP-Cu	Nanoindentation	12	N/A	17
CNM	$PAH(SNP/CS)_{4.5}$	Buckling	4.8	N/A	18
	$PAH(NFC/CS)_{9.5}$	Buckling	3.5	N/A	
	Polystyrene	Buckling	3.6	N/A	19
	CNC/PVOH	Buckling	21	N/A	20
	$(PAH\ 1\ M\text{-}CNF\ 0\ mM)_4$	Buckling	9.3	N/A	21
	$(PAH\ 0\ M\text{-}CNF\ 12\ mM)_4$	Buckling	5.5	N/A	
	CBPS-CNM	Buckling	14.7	N/A	22
	9G9	Buckling	15.4	N/A	23
	Decacyclene	Nanoindentation	6	N/A	24

Supplementary References

1 Huang, W.-H., Liu, S., Zavalij, P. Y. & Isaacs, L. Nor-seco-cucurbit [10] uril exhibits homotropic allosterism. *J. Am. Chem. Soc.* **128**, 14744-14745 (2006).

2 Frisch, M. *et al.* Gaussian 16 rev. c. 01, wallingford, ct. *Wallingford, CT* (2016).

3 Mücke, D. *et al.* Understanding the electron beam resilience of two-dimensional conjugated metal–organic frameworks. *Nano Lett.* **24**, 3014-3020 (2024).

4 Sahabudeen, H. *et al.* Wafer-sized multifunctional polyimine-based two-dimensional conjugated polymers with high mechanical stiffness. *Nat. Commun.* **7**, 13461 (2016).

5 Liu, K. *et al.* A two-dimensional polyimide-graphene heterostructure with ultra-fast interlayer charge transfer. *Angew. Chem. Int. Ed.* **60**, 13859-13864 (2021).

6 Iguiñiz, N., Frisenda, R., Bratschitsch, R. & Castellanos-Gomez, A. Revisiting the buckling metrology method to determine the Young's modulus of 2D materials. *Adv. Mater.* **31**, 1807150 (2019).

7 Zeng, Y. *et al.* Irreversible synthesis of an ultrastrong two-dimensional polymeric material. *Nature* **602**, 91-95 (2022).

8 Yang, Y. *et al.* Elastic films of single-crystal two-dimensional covalent organic frameworks. *Nature* **630**, 878-883 (2024).

9 Fang, Q. *et al.* Superior mechanical properties of multilayer covalent-organic frameworks enabled by rationally tuning molecular interlayer interactions. *Proc. Natl. Acad. Sci. U.S.A.* **120**, e2208676120 (2023).

10 Hao, Q. *et al.* Confined synthesis of two-dimensional covalent organic framework thin films within superspreading water layer. *J. Am. Chem. Soc.* **140**, 12152-12158 (2018).

11 Dey, K., Bhunia, S., Sasmal, H. S., Reddy, C. M. & Banerjee, R. Self-assembly-driven nanomechanics in porous covalent organic framework thin films. *J. Am. Chem. Soc.* **143**, 955-963 (2021).

12 Miller, K. A. *et al.* High-strength, microporous, two-dimensional polymer thin films with rigid benzoxazole linkage. *ACS Appl. Mater. Interfaces* **14**, 1861-1873 (2022).

13 Zhao, Z. *et al.* Horizontally arranged zinc platelet electrodeposits modulated by fluorinated covalent organic framework film for high-rate and durable aqueous zinc ion batteries. *Nat. Commun.* **12**, 6606 (2021).

14 Zeng, Z., Flyagina, I. S. & Tan, J.-C. Nanomechanical behavior and interfacial deformation beyond the elastic limit in 2D metal–organic framework nanosheets. *Nanoscale Adv.* **2**, 5181-5191 (2020).

15 Hermosa, C. *et al.* Mechanical and optical properties of ultralarge flakes of a metal–organic framework with molecular thickness. *Chem. Sci.* **6**, 2553-2558 (2015).

16 Huang, C. *et al.* Synthesizing conductive metal–organic framework nanosheets for high-performing chemiresistive sensors. *ACS Appl. Mater. Interfaces* **17**, 18771-18780 (2025).

17 Sahabudeen, H., Zhang, Q., Liu, Y., Heuchel, M. & Machatschek, R. Mechanistic insights into the deformation and degradation of a 2D metal organic framework. *npj 2D Mater. Appl.* **7**, 25 (2023).

18 Johansson, E. & Wågberg, L. Tailoring the mechanical properties of starch-containing layer-by-layer films. *Colloids Surf. A: Physicochem. Eng. Asp.* **394**, 14-22 (2012).

19 Stafford, C. M. *et al.* A buckling-based metrology for measuring the elastic moduli of polymeric

thin films. *Nat. Mater.* **3**, 545-550 (2004).

20 Niinivaara, E., Desmaisons, J., Dufresne, A., Bras, J. & Cranston, E. D. Thick polyvinyl alcohol films reinforced with cellulose nanocrystals for coating applications. *ACS Appl. Nano Mater.* **4**, 8015-8025 (2021).

21 Azzam, F. *et al.* Relationship between Young's modulus and film architecture in cellulose nanofibril-based multilayered thin films. *Langmuir* **33**, 4138-4145 (2017).

22 Ai, M. *et al.* Carbon nanomembranes (CNMs) supported by polymer: mechanics and gas permeation. *Adv. Mater.* **26**, 3421-3426 (2014).

23 Markutsya, S., Jiang, C., Pikus, Y. & Tsukruk, V. V. Freely suspended layer-by-layer nanomembranes: testing micromechanical properties. *Adv. Funct. Mater.* **15**, 771-780 (2005).

24 van der Ham, A. *et al.* Freestanding non-covalent thin films of the propeller-shaped polycyclic aromatic hydrocarbon decacyclene. *Nat. Commun.* **13**, 1920 (2022).
